# The potential of H5N1 viruses to adapt to bovine cells varies throughout evolution

**DOI:** 10.1038/s41467-025-67234-1

**Published:** 2025-12-15

**Authors:** Matthew L. Turnbull, Mohammad Khalid Zakaria, Nicole S. Upfold, Siddharth Bakshi, Callum Magill, Udeet Ranjan Das, Andrew T. Clarke, Laura Mojsiejczuk, Vanessa Herder, Kieran Dee, Nancy Liu, Monika Folwarczna, Georgios Ilia, Wilhelm Furnon, Verena Schultz, Hanting Chen, Ryan Devlin, Jack McCowan, Alex L. Young, Wai-Wai Po, Katherine Smollett, Muhammad Ahsan Yaseen, Rebecca Ross, Avanti Bhide, Bianca van Kekem, Ron A. M. Fouchier, Ana da Silva Filipe, Munir Iqbal, Ed Roberts, Joseph Hughes, Dirk Werling, Pablo R. Murcia, Massimo Palmarini

**Affiliations:** 1 MRC-University of Glasgow Centre for Virus Research, Glasgow, UK; 2 Department of Viroscience, Erasmus Medical Centre, Rotterdam, The Netherlands; 3 CRUK Scotland Institute, Glasgow, UK; 4 Faculty of Veterinary Medicine, University of Teramo, Loc. Piano D’Accio, Teramo, Italy; 5 Istituto Zooprofilattico Sperimentale dell’ Abruzzo e Molise “G. Caporale”, Teramo, Italy; 6 The Pirbright Institute, Woking, UK; 7 Department for Pathobiology and Population Sciences, Centre for Vaccinology and Regenerative Medicine, Royal Veterinary College, London, UK

**Keywords:** Influenza virus, Microbiology

## Abstract

Avian influenza H5N1 clade 2.3.4.4b viruses caused a global panzootic and, unexpectedly, widespread outbreaks in dairy cattle, therefore representing a pandemic threat. To inform control strategies, it is critical to determine whether the potential to adapt to bovine cells is a general feature of H5N1 viruses, is specific to viruses of clade 2.3.4.4b, or narrowly restricted to some genotypes within this clade. Using a large panel of recombinant viruses representing >60 years of H5N1 history and other IAVs for comparison, we demonstrate replicative fitness in bovine cells is: (i) highly variable across 2.3.4.4b genotypes, (ii) limited in viruses predating the global expansion of this clade, (iii) determined by the internal gene cassette, and (iv) not restricted to udder epithelial cells. Mutations in the PB2 polymerase subunit emerge as key determinants of adaptation, although their phenotypic effects are context dependent. Bovine B3.13 and some avian genotypes exhibit enhanced modulation of bovine interferon-induced antiviral responses, determined by at least PB2, nucleoprotein, and the non-structural protein NS1. Our results highlight the polygenic nature of IAV host range, and reveal that the replication fitness in bovine cells, and likely their potential to adapt to cattle, varies greatly during the evolutionary trajectory of H5N1 viruses.

## Abstract (web summary)

H5N1 avian influenza viruses caused an outbreak in dairy cattle. We show that the potential for avian viruses to replicate in cow cells varies across H5N1 evolution, suggesting that the risk of spillover into mammals differs between variants.

## Introduction

Influenza A viruses (IAV) represent a major threat to human and animal health. Wild aquatic birds are the main natural reservoir of IAV as they maintain multiple antigenically and genetically distinct subtypes of their external glycoproteins^1^ (17 hemagglutinins, HA, and 9 neuraminidases, NA). Humans, pigs, and horses instead are hosts to a limited number of IAV subtypes, originating from birds, which circulate in an endemic cycle within each species after pandemic/panzootic emergence (e.g. H1N1 and H3N2 in humans)^1^.

Certain avian IAV subtypes including H5Nx (where x represents distinct N subtypes), H7N9, H9N2, H10Nx and others, have occasionally infected some mammalian species including humans. These spillover events almost never lead to documented mammal-to-mammal transmission chains, but they nevertheless represent a potential pandemic risk, considering that each of the four influenza human pandemics that have occurred since 1918 were caused by viruses carrying at least some genomic segments from avian influenza IAV^2–6^.

Within this context, highly pathogenic H5N1 viruses are of particular concern as they can cause devastating outbreaks in poultry and have spilled over repeatedly into humans and other mammals^7^. Since 2021, H5N1 viruses within clade 2.3.4.4b have spread worldwide at unprecedented levels^8,9^, infecting a variety of mammalian species such as minks, skunks, raccoons, cats, bears, foxes^10–13^ and aquatic mammals including harbour seals and sea lions^14–17^. As these viruses spread, they reassorted with co-circulating low pathogenic avian IAV resulting in over 80 genotypes to date that differ from each other in the constellation of their “internal” genomic cassette (i.e. the viral genome segments encoding all the viral proteins but HA and NA). Unexpectedly, in March 2024, H5N1 (B3.13 genotype) caused an influenza outbreak in dairy cattle in the US^18^. Until these outbreaks, cows had not been regarded as natural hosts of IAVs despite previous studies detecting anti-IAV antibodies in cattle sera, and experimental inoculations showing that cows are susceptible to both human and avian IAVs^19^ (reviewed in ref. ^20^). Affected cows showed clinical signs such as fever, depression, drop in milk production and mastitis, but only rarely signs of mild respiratory infections. Indeed, high levels of infectious virus could be detected consistently in the milk of infected animals^18^. The B3.13 genotype emerged after a European-origin 2.3.4.4b (EA-2020-C) entered North America in 2021^9,21^, and acquired PB2, PB1, NP and NS segments via sequential reassortment events with local low pathogenic avian influenza viruses (LPAIV)^21–24^. Phylogenetic analysis supports the notion that a single introduction event from wild birds into cattle occurred in late 2023 or early 2024 and this has subsequently spread to multiple dairy farms across 17 US states^24–26^. At the beginning of 2025, the emergence of a distinct genotype (D1.1) was reported in dairy cattle in Nevada and Arizona^27^. In the case of the D1.1 outbreak, a European-origin 2.3.4.4b virus had instead acquired the PB2, PA, NP and NA segments from local LPAIV via reassortment^23,28^. Bovine H5N1 spilled over to humans (70 confirmed human cases in the US as of August 2025^25^), in addition to cats and other small mammals in the vicinity of affected farms. Furthermore, a 2.3.4.4b virus of a genotype ancestral to the B3.13 dairy cattle virus (B3.6) caused a limited outbreak in goats^29^.

There are several known barriers to avian IAV adaptation in mammalian cells (reviewed in ref. ^30^), and cellular receptor specificity is a critical one. Avian HAs preferentially bind glycans that terminate with an α2,3-sialic acid, which are abundant in the gastroenteric tract of birds, while human-adapted IAV HAs preferentially bind to the α2,6-sialic acid conformation, which is abundant in the upper respiratory tract of humans and other mammals^31–35^. Several recent studies have focused on the bovine H5N1 virus glycoproteins and their receptor preference and tissue tropism^36–45^. α2,3- linked glycans are abundant in the udder tissue of dairy cattle^37–39^. The HA of 2.3.4.4b H5N1 virus associated with both bovine and human infections of the recent dairy cattle outbreak appears to retain a preference for ‘avian like’ α2,3-sialyl glycans^40–42^, although ability to bind α2,6-sialyl glycans to an extent has also been reported^36^. Importantly, only a single amino acid change appears to allow the HA from an IAV isolated from a human dairy farm worker to bind α2,6-sialic receptors efficiently^43,44^, highlighting the potential for human-adaptation. Other glycoprotein-associated determinants hampering avian IAV transmission to mammals include pH sensitivity of HA, and the length of the NA glycoprotein stalk^46,47^.

Viral proteins encoded by the internal genomic segments (i.e. the PB2, PB1, PA, NP, M, and NS) also constitute key molecular determinants of virus host range. For example, polymerase complexes of avian IAV exhibit reduced efficiency in utilising mammalian ANP32 proteins, which are essential cofactors for IAV replication^48–53^, and the nuclear import machinery^54^. The nucleoprotein (NP) of avian viruses is the target of interferon stimulated genes myxovirus resistance protein 1 (MxA in humans, Mx1 in other vertebrates) and butyrophilin subfamily 3 member A3 (BTN3A3), which human-adapted IAVs evolved to escape^55–60^. The non-structural protein 1 (NS1) and PA-X are multifunctional proteins, that counteract the IFN response and are also associated with host-range^55,61,62^.

The ability of avian IAVs to replicate efficiently in mammalian cells and evade host restriction factors are crucial initial components of successful spillover and adaptation events. Following the cattle outbreak, a limited number of studies have experimentally shown that certain avian-derived H5Nx clade 2.3.4.4b viruses can replicate in bovine cells and tissues, albeit with lower efficiency compared to cattle-derived B3.13 viruses^63–65^. In contrast, in-vivo infection of dairy cows showed that the circulating H5N1 genotype euDG can induce levels of infection and disease similar to those induced by B3.13^66^. Overall, these studies sampled a small subset of clade 2.3.4.4b viruses.

Here, we systematically characterised the phenotypes of over 80 recombinant viruses, representing 15 distinct H5Nx genotypes spanning over 60 years of evolution, using a variety of in vitro assays to determine whether replicative fitness and innate immune evasion in bovine cells are a generalised feature of this group of viruses, traits specific to viruses within the 2.3.4.4b clade, or are even more narrowly restricted to particular genotypes within this clade. Overall, our data indicates that the replication fitness of H5N1 viruses in bovine cells varies throughout evolution and is governed by multiple internal genomic segments.

## Results

### Reassortants generated in this study

**Fig. 1:**
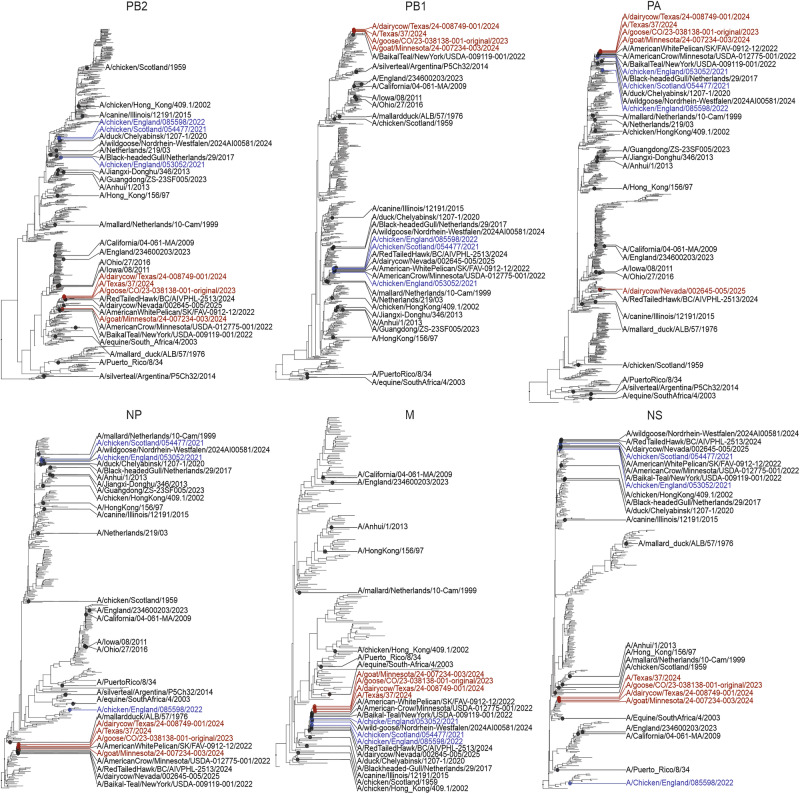
Global phylogenies of IAV sequences used in this study. Global maximum likelihood phylogenetic trees of nucleotide sequences from the internal segments of IAVs used in this study. Tips are annotated by strain names and correspond to viruses evaluated experimentally. Viruses of interest belonging to the European and North American 2.3.4.4b clade are coloured blue and red, respectively.

To investigate the molecular determinants of H5N1 adaptation to the bovine host, we first generated a set of 23 2:6 reassortants carrying the glycoproteins (HA and NA) of the laboratory-adapted H1N1 A/Puerto Rico/8/34 (PR8) strain, and the six internal genomic segments of various H5N1 viruses (Fig. 1). Using reassortant viruses carrying identical glycoproteins allowed us to directly scrutinise the role of the viral proteins encoded by the internal gene cassette in adaptation to bovine cells.

Each reassortant used in this study is termed using the prefix “r” followed by an abbreviated name of the virus in question (Tables S1 and S2). For example, r-Bovine-B3.13 possesses the internal genes from one of the earliest sampled H5N1 bovine influenza sequences (sample acquired from a dairy cow on 20^th^ March 2024 in Texas). r-Texas/37-B3.13 is a similar, but distinct, B3.13 that infected a dairy farm worker and likely derives from a common ancestor of the lineage that has spread in cattle^24^. r-Bovine-D1.1 contains the internal genes of a distinct H5N1 outbreak of genotype D1.1 that was sampled in January 2025. r-Goat-B3.6 possesses the internal genes from the 2.3.4.4b virus isolated in goats (B3.6 genotype)^29^. We also generated reassortants with the internal genes of several other H5N1 2.3.4.4b genotypes isolated from birds from Europe and North America, including among those a reassortant representing the European virus that entered North America (r-EA-2020-C). In addition, we also generated H5 viruses predating the global expansion of the H5N1 2.3.4.4b clade. These include the first sequenced HPAI H5N1 virus from 1959 (r-CHK/Scot/59); a non-GsGd lineage of H5N2 from 1976 (r-Mld/76); a clade 4 H5N1 from 2002 (r-CHK/02); an early 2.3.4.4b H5N6 from 2017 (r-BHG/17). For comparative purposes we included reassortants of the prototypic pandemic human 2009 virus (r-pdm09(H1N1)), and other mammalian adapted viruses (complete list of viruses used in Table S1).

We also generated 4:4 reassortants containing HA, NA, M and NS from PR8 and only the polymerase complex (PB2, PB1, PA and NP) from certain viruses of interest for specific assays (Table S2); these include some of the viruses indicated above, and others of avian [r-Arg(H4N2), r-Chn/23(H3N8), r-Mld/99(H1N1), r-Nld(H7N7), r-Chn/13(H10N8)], or swine [r-Iowa/11(H2N2v), r-Ohio/16(H3N2v)] origin. It should be noted that the virus from which the reverse genetics constructs were derived for Mld/99(H1N1) had been previously passaged in pig cells^67^. All reassortant viruses were rescued in HEK-293T cells, or in HEK-293T-Gg.ANP32A cells (293 T cells expressing chicken ANP32) for those that could not be rescued in the parental cell-line (Table S1). Viral stocks were propagated and titrated in MDCK (or MDCK-Gg.ANP32A cells, Table S1) and fully sequenced as described in Methods.

### Viruses carrying internal genes derived from North American 2.3.4.4b ruminant viruses display the fastest replication kinetics in bovine cells

**Fig. 2:**
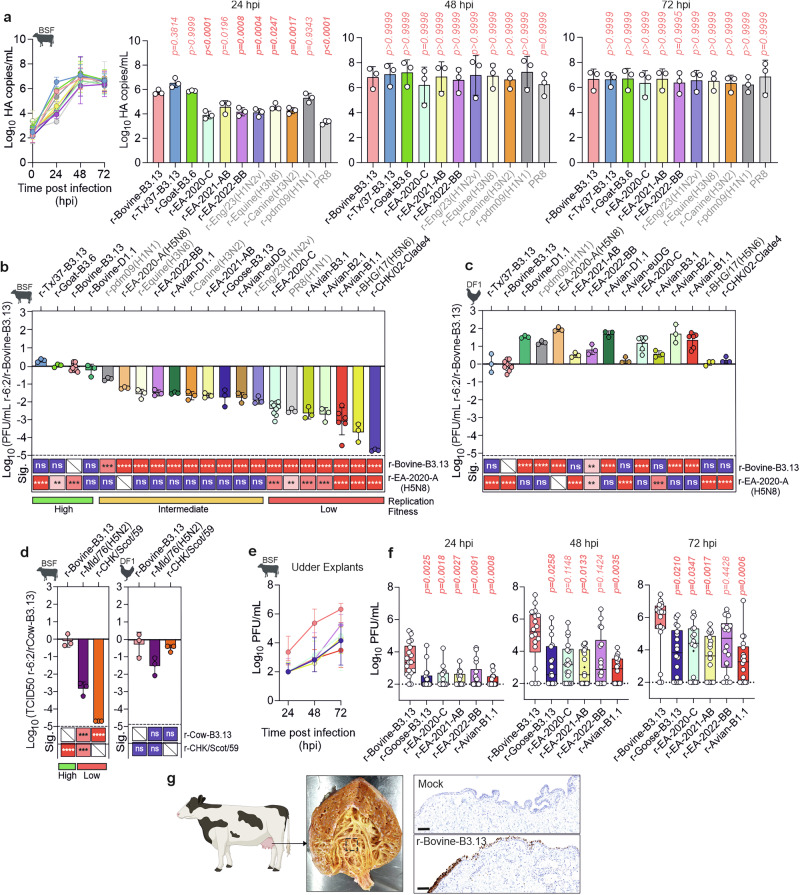
Replication of 2.3.4.4b and other IAV reassortant viruses in bovine cells and udder tissue. **a** Viral RNA in supernatants of bovine skin fibroblasts (BSF) infected with 0.0005 genome copies/cell, quantified by RT-qPCR. **b–d** Infectious titres at 24 hpi in BSF (**b**) and chicken embryonic fibroblasts (DF1) (**c**) infected with 0.001 PFU/cell, measured by plaque assay on MDCK cells (**b, c**) or TCID50 on DF1 cells (**d**). **e, f** Viral titres from bovine udder explants infected with 1000 PFU/explant, quantified by plaque assay on MDCK cells. **g** Immunohistochemistry of r-Bovine-B3.13- or mock-infected udder explants showing IAV nucleoprotein-positive cells (brown) in the epithelial layer. In (**a–d**), data are mean ± s.d. from three biological replicates (n = 3), except in **b** and **c**, where r-Bovine-B3.13 (n = 6), r-EA-2020-C (n = 9 in (**b**), n = 6 in (**c**)) and r-Avian-B1.1 (n = 6); in (**a**) data points are mean of two technical duplicates. Data were Log-transformed (**a**) or normalised to r-Bovine-B3.13 (**b–d**). Statistics were determined by one-way ANOVA with Tukey’s post-hoc test (two-tailed; α = 0.05). Comparisons are shown relative to r-Bovine-B3.13(H5N1) in all panels, with additional comparisons to r-EA-2020-A(H5N8) or r-CHK/Scot/59 in **b–d**. In **e, f**, each data point represents one independently infected tissue explant (n = 18). Data were log-transformed and statistical comparisons were determined using Kruskal-Wallis test with Dunn’s multiple comparisons (two-tailed; α = 0.05). **e** Shows data medians with interquartile range (error bars). In **f**, box-and-whisker plots show the median (central line), interquartile range (box) and full data range (whiskers). Individual data points are overlaid. Significance: ns = p > 0.05; * = p ≤ 0.05; ** = p ≤ 0.01; *** = p ≤ 0.001; **** = p ≤ 0.0001. H5N1 viruses are shown in black, non-H5N1 IAVs in grey. Data in **g** are representative of one experiment, validated by five optimisation runs with varying primary antibody dilutions showing the same result. (**a–e**) and (**g**) images created in BioRender. Bakshi, S. (2025) https://BioRender.com/vtz04mr.

We first compared the replication kinetics of the panel of recombinant viruses in an IFN-competent cell line that we derived from bovine skin fibroblasts (BSF)^68^. Based on the in vitro phenotype in these cells, we could divide the viruses tested into three broad groups with high, intermediate, or low replicative fitness. r-Bovine-B3.13, r-Texas/37-B3.13, r-Goat-B3.6, and r-Bovine-D1.1 displayed the fastest replication kinetics, reaching higher titres at 24 hours post infection (hpi) than the other avian- and mammalian-origin IAVs tested. This included avian viruses ancestral to the B3.13 and D1.1 cattle outbreaks, as well as several distinct avian IAV genotypes (Fig. 2a, b & Supplemental Fig. S1a, b). Reassortant viruses exhibiting an intermediate replication phenotype included avian viruses belonging to genotypes EA-2020-A, EA-2022-BB, D1.1, EA-2021-AB, B3.13, and euDG, which replicated as well as the mammalian-adapted viruses we tested (r-equine (H3N8), r-canine (H3N2), r-Eng/23 (H1N2v)). Of note, many of these viruses have been shown already to cross the species barrier. EA-2022-BB has caused outbreaks in fur mammals^69,70^, B3.13 and D1.1 in dairy cattle, and euDG has been shown to experimentally infect dairy cattle and replicate to high titres^66^. These virus genotypes belong to clade 2.3.4.4b and have been detected in avian hosts within the past 3 years. Reassortant viruses that replicated relatively poorly in BSFs included reassortants derived from some 2.3.4.4b genotypes (e.g. EA-2020-C, B2.1, B3.1, r-BHG/17 (H5N6)) and others that predate the origin of H5N1 2.3.4.4b viruses (i.e. r-CHK/Scot/59(H5N1), r-Mld/76(H5N2), r-HK/CHK/02-Clade4(H5N1) (Fig. 2b, d).

Importantly, the avian-origin recombinant viruses of the panel replicated as efficiently or better than r-Bovine-B3.13 in chicken DF-1 fibroblast cells (Fig. 2c, Fig. S1c, d), and also relatively well in the permissive MDCK cell line (Fig. S2a), suggesting this is a host species-specific phenotype and that intrinsic replication properties of the recombinant viruses (such as segment compatibility and RNA packaging) were not obviously attenuated. There seemed to be an inverse relationship between replication fitness in bovine and avian cells (i.e. ruminant viruses replicated well in bovine cells and less efficiently in avian cells and vice versa relative to the panel of avian-derived viruses). The only exception to this trend was r-Bovine-D1.1, which replicated to high levels in bovine and avian cells.

The overall trends observed in BSF were also observed in primary nasal fibroblasts (BNF) isolated from bovine nasal tissue received from an abattoir (Fig. S2b), suggesting this was not a phenotype specific to skin fibroblast cells. Importantly, the replication advantage observed for r-Bovine-B3.13, a representative ruminant-origin IAV, was maintained also in ex vivo bovine udder explant tissue from multiple donors. In contrast, avian-derived recombinant viruses showed reduced replication, supporting the reliability of the phenotype observed in the bovine skin fibroblast model (Fig. 2e, f). This trend was also consistent across a range of multiplicities of infection when compared to the earlier European r-EA-2020-C virus which entered North America (Fig. S3a). Of note, our 2:6 viruses appeared to replicate preferentially in epithelial cells of the cistern of the mammary glands (Fig. 2g, Fig. S3b, c) as assessed by immunohistochemistry of fixed tissues. Although clear trends were observed, the data showed considerable variability. To ensure statistical robustness, we conducted experiments using 18 replicates derived from tissue from six different donors. This variability is largely attributed to the technical challenges in obtaining explants with a homogenous size of cisternal epithelium.

Overall, these data suggest that the internal genomic segments of viruses associated with recent outbreaks in ruminants in North America provide a replication advantage in bovine cells relative to other diverse avian- and mammalian-origin IAVs. These viruses contain internal genes derived from three distinct genotypes (B3.6, B3.13 and D1.1), suggesting that more than one constellation of internal genomic segments can confer enhanced replication fitness in a bovine host. While many of the avian clade 2.3.4.4b H5N1 viruses tested also replicate efficiently in bovine cells, their replication levels are generally lower than those of the outbreak-associated strains. This suggests that although efficient replication is not unique to viruses like B3.13 and D1.1, the replication advantage observed in outbreak strains remains distinct and measurable. In contrast, the older avian H5N1 viruses predating the clade typically showed poor replication in bovine cells, further highlighting the variability in host adaptation across lineages. These findings support the notion that replication potential in bovine cells varies widely across H5N1 lineages, clades, and genotypes, and it is independent of the udder microenvironment.

### Bovine-specificity of B3.13, D1.1 and B3.6 viruses does not directly correlate to high polymerase activity in human cells

**Fig. 3:**
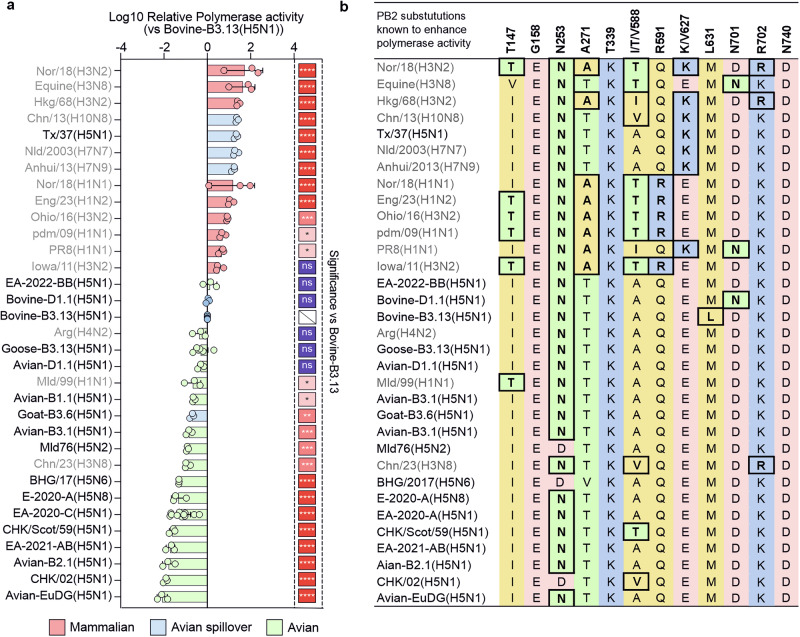
Polymerase activity of diverse IAV strains in human cells. **a** Minireplicon assays in HEK293T cells at 24 hpt to assess polymerase activity of a broad panel of Influenza A viruses. Data are mean +/- s.d. of three biological replicates (n = 3), except for Bovine-B3.13 (n = 15), Goose-B3.13 (n = 6) and EA-2020-C (n = 12). Each data point corresponds to the mean of two technical duplicates per biological replicate. All values were normalised to r-Bovine-B3.13, log-transformed, and statistical comparisons between all data were determined using one-way ANOVA with Dunnett’s multiple comparison test (two-tailed; α = 0.05), with comparisons shown relative to r-Bovine-B3.13. Statistical symbols: ns = p > 0.05; * = p ≤ 0.05; ** = p ≤ 0.01; *** = p ≤ 0.001; **** = p ≤ 0.0001. H5N1 virus polymerases are shown in black, non-H5N1 IAVs in grey. **b** Table of PB2 residues previously implicated in increased polymerase activity. Black boxes indicate residues associated with increased polymerase activity described in earlier studies^48,71–74,103,131–135^.

The activity of the viral polymerase complex (PB1, PB2, PA and NP) is a critical determinant of IAV within-host fitness and therefore central for virus tropism and adaptation. As the viruses we assessed exhibited different replication kinetics, we performed standard minireplicon assays in human 293 T cells to evaluate the specific contribution of the polymerase to the observed phenotype in mammalian cells. We tested the polymerase complexes of all the viruses of the 2:6 panel described above, as well as a variety of other control viruses (outlined in Tables S1–S3), to obtain a comprehensive and comparative overview of their activity in human cells (Fig. 3a).

The highest activity was reached with the polymerases from IAVs of human-origin or those associated with human spillover events. Among the top 13 ranked polymerases, all but an equine-origin virus and the laboratory-adapted PR8 were derived from human-endemic or spillover viruses of avian- or swine-origin. These viruses possess mammalian-adaptative mutations in PB2 including the well-described 627 K, 701 N and 591 R substitutions, among others (Fig. 3b)^48,71–73^.

Bovine-derived polymerases displayed intermediate activity (Fig. 3a), while most avian-derived polymerases exhibited, as expected, the lowest activity in mammalian cells. The sequence of PB2 derived from r-Bovine-B3.13 H5N1 does not possess any major known PB2 adaptive mutations, with the exception of M631L which has been reported to boost polymerase activity of a mouse-adapted H10N7 virus and has recently been shown to contribute to enhanced activity of a human-derived B3.13 polymerase in human cells^7,74,75^.

Some avian IAV polymerases, including that of EA-2022-BB, which ranked highest among avian-origin polymerases, exhibited activity levels comparable to those of bovine-adapted viruses. Notably, the EA-2022-BB genotype has been implicated in outbreaks among farmed fur mammals in Europe^69,70^. Other relatively high-ranking avian polymerases included Avian-D1.1 and Goose-B3.13, both closely related to viruses that emerged in cattle. The goat-derived polymerase displayed lower activity than other avian-origin polymerases, including Avian-D1.1 and Goose-B3.13.

Taken together, these data highlight that adaptation of certain H5N1 viruses to bovine cells is also correlated with a relative enhancement of polymerase activity in human cells, although this does not reach the levels of human-adapted viruses. Importantly, polymerases from some specific avian viruses also possess activity in human cells similar to bovine-adapted viruses. The data also suggest that polymerase activity alone is not necessarily the only predictor of virus fitness in replication assays, and that additional genomic segments may compensate for suboptimal polymerase activity.

### r-Bovine-B3.13 is more virulent than ancestral r-EA-2020-C in mice

We next inoculated C57BL/6 mice with 100 PFU of either r-Bovine-B3.13, r-EA-2020-C or PR8 as control, to test whether the internal genes of bovine B3.13 virus were sufficient to drive viral spread in respiratory tissues and determine virulence in this widely used experimental model for IAV^76^. As expected, PR8 (a mouse-adapted laboratory strain) caused severe weight loss in infected animals, while the weight of mice infected with r-EA-2020-C was essentially unaffected. Mice infected with r-Bovine-B3.13 displayed an intermediate phenotype showing weight loss but not as severe as those infected with PR8 (Fig. S4a). Both quantification of NP in the lungs of infected mice euthanised at 3 days post-infection (dpi) and T-cell infiltrates (CD3) in mice euthanised at 8 dpi displayed similar trends, with higher values in mice infected with PR8, followed by those infected with r-Bovine-B3.13 and then r-EA-2020-C, although differences were not statistically significant due to variability within groups (Fig. S4b–d). Thus, the internal genes of bovine B3.13, unlike those of the avian EA-2020-C genotype, are sufficient to induce some pathology in mice, although not as pronounced as those induced by wild type B3.13 in published studies^77,78^.

### Synergistic interactions among internal genomic segments of B3.13 viruses contribute to their higher replication fitness in bovine cells

**Fig. 4:**
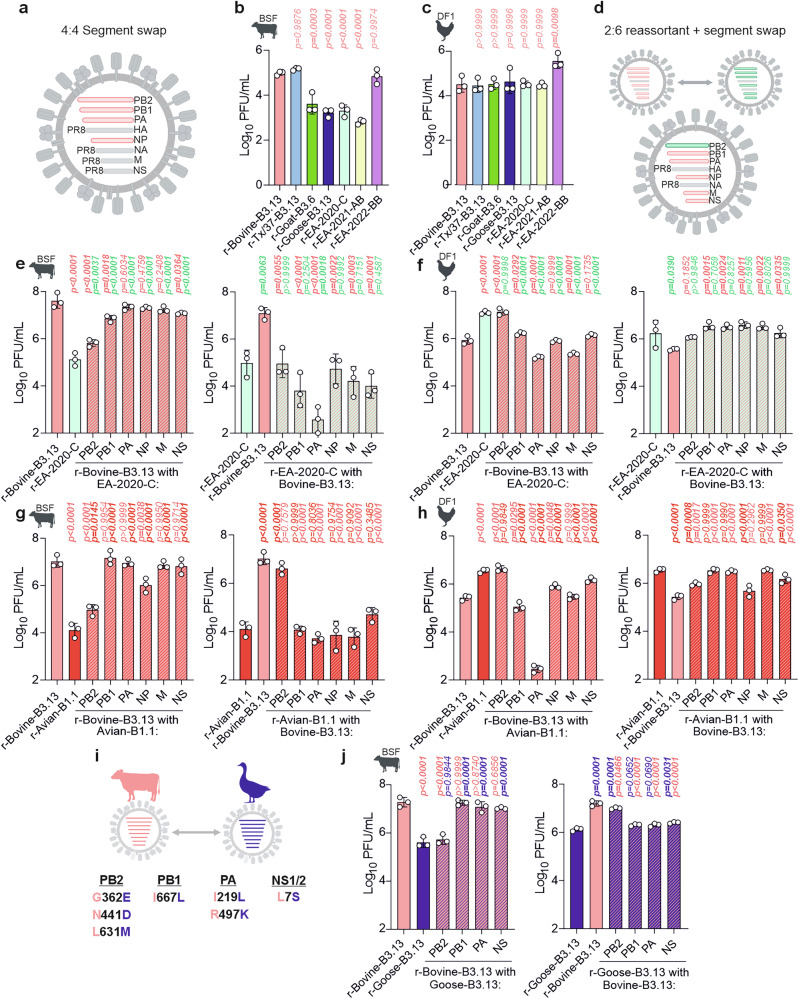
Segment-specific contribution of B3.13 internal genes to virus replication. **a** Schematic of 4:4 reassortant virus containing segments 1,2,3 and 5 from an H5N1 virus in a PR8 backbone. **b, c** Replication of 4:4 recombinant viruses in BSF (**b**) or DF1 (**c**) cells for 24 hpi. **d** Schematic of 2:6 viruses used in **e–h**, with internal gene segments reciprocally exchanged between r-Bovine-B3.13 and either r-EA-2020-C or r-Avian-B1.1. **e–h** Replication of 2:6 reciprocal viruses in BSF (**e, g**) and DF1 (**f, h**) cells at 24 hpi. **i** Amino acid changes between r-Bovine-B3.13 and r-Goose-B3.13. **j**, Replication of 2:6 reciprocal viruses between r-Bovine-B3.13 and r-Goose-B3.13 in BSF cells. An MOI of 0.001 PFU/cell was used in all infections; virus released into the supernatant at 24 hpi was titrated by plaque assay on MDCK cells. Data are mean +/- s.d. of three biological replicates (n = 3); Values were log-transformed and multiple comparisons between all viruses were determined by one-way ANOVA with Tukey’s test (two-tailed; α = 0.05); only comparisons relative to controls are shown. P-values in bold indicate statistical significance. **a–j** Created in BioRender. Bakshi, S. (2025) https://BioRender.com/bolea9d.

Our data suggested that polymerase activity measured in minireplicon assays is not a stand-alone predictor of replicative fitness of live viruses in bovine cells. To directly assess the role of the polymerase complex during viral replication, we infected bovine (BSFs) and avian (DF1) cells with PR8-recombinant viruses harbouring the polymerase and NP genes from select viruses (referred to as 4:4 viruses). When 4:4 viruses were compared in BSF cells, the replicative fitness of the viruses in the panel was different from their fitness as 2:6 viruses (which harboured their cognate M and NS segments, Fig. 4a–c). Whilst r-Bovine-B3.13 and TX/37-B3.13 were the fittest in this panel of 4:4 viruses in BSF cells, there was no longer a clear advantage over the avian r-EA-2022-BB 4:4 virus, as was observed in the context of the 2:6 assays (Fig. 2a, b). Furthermore, the r-Goat-B3.6 4:4 virus, which displayed one of the fastest replication kinetics as a 2:6 virus (Fig. 2a, b), replicated at similar levels to those observed by avian-origin viruses r-EA-2020-C and r-Goose-B3.13. Importantly, all the 4:4 viruses replicated as well as, or in the case of r-EA-2022-BB better than, the r-Bovine-B3.13 4:4 virus in chicken DF-1 cells. Overall, while these findings are consistent with the results from human polymerase assays, they also suggest that M and/or NS play an important role in virus replication in bovine cells.

To further dissect the role(s) of individual segments, we generated single-segment reassortants between r-Bovine-B3.13 and r-EA-2020-C (Fig. 4d), a representative of the European genotype that first entered North America. There are multiple changes across the genome between these two viruses (see Supplementary Data 1 for an alignment of all internal gene proteins from avian H5Nx viruses used in this study relative to r-Bovine-B3.13). Of the non-spliced products, the NP protein has the fewest number of changes (3 substitutions) while each of the polymerase subunits has at least 10 substitutions each. When individual segments of the bovine virus were replaced with those from avian r-EA-2020-C, there was a significant reduction in titre for the PB2, PB1 and NS segment reassortants, with PB2 causing the largest decrease relative to the bovine control (Fig. 4e). None of these individual swaps reduced the titre to the level of the r-EA-2020-C control, however. In the reciprocal reassortant viruses, none of the internal segments from the bovine B3.13 reassortant increased replication of r-EA-2020-C virus in bovine cells. Indeed, only when four segments of bovine B3.13 (PB2, PB1, NP and NS) were introduced together into EA-2020-C, reflecting the four segments derived from North American LPAI during the emergence of B3.13, was there a clear effect on the replication (Fig. S5). Importantly, reassortant viruses that were attenuated relative to the r-Bovine-B3.13 control in bovine cells, replicated to high titres in chicken cells, suggesting these viruses did not have an obvious intrinsic defect (such as genome packaging with the PR8 HA and NA segments) (Fig. 4f).

We next rescued a set of reassortants between r-Bovine-B3.13 and r-Avian-B1.1, a virus that replicated particularly poorly in BSF cells and udder tissue (Fig. 2b) and is a distinct American genotype acquiring PB2, PB1 and NP from local LPAIV^79^. As with EA-2020-C, there were multiple changes across the genome relative to r-Bovine-B3.13 (Supplemental File 1) with PB2 having the greatest number of substitutions (13) and NP the fewest (4) among the non-spliced genes. In these reassortants, the avian B1.1 PB2 significantly reduced the replication of r-Bovine-B3.13 in BSF cells, although not to the level observed for the r-Avian-B1.1 control. However, B1.1. PB2 did not affect titres in chicken DF-1 cells (Fig. 4g, h). The avian B1.1 NP segment also modestly reduced the replication of r-Bovine-B3.13. In the reciprocal setup, bovine PB2 rescued r-Avian-B1.1 replication to levels comparable to the r-Bovine-B3.13 control, suggesting a more dominant role for PB2 in this virus context.

Finally, we explored the contribution of individual segments to the phenotypic differences observed between bovine B3.13 and the reassortant derived from the closest sequenced B3.13 virus isolated from birds (r-Goose-B3.13). As there are only a small number of amino acid changes in PB2, PB1, PA and NS (Fig. 4i), only these segments were swapped between viruses. In bovine cells, recombinant viruses with either goose-B3.13 PB1, PA or NS in r-Bovine-B3.13 reached titres similar to r-Bovine-B3.13. Importantly, the r-Bovine-B3.13 reassortant bearing the PB2 segment from goose-B3.13 fully recapitulated the replication phenotype of r-Goose-B3.13 (Fig. 4j). These data suggest that PB2 adaptive mutations played a particularly important role in improving virus replication after emergence in dairy cattle, in accordance with previous studies and observations^75,80^.

Overall, our segment swap data highlight that efficient replication in bovine cells requires multiple adaptive mutations, likely due to the presence of multiple, distinct bovine barriers to avian influenza viruses. The number, position, and nature of adaptive mutations are likely to vary depending on the genomic constellation of the virus genotype.

### PB2 M631L is an important adaptive mutation for bovine B3.13 virus in bovine cells but is context dependent

**Fig. 5:**
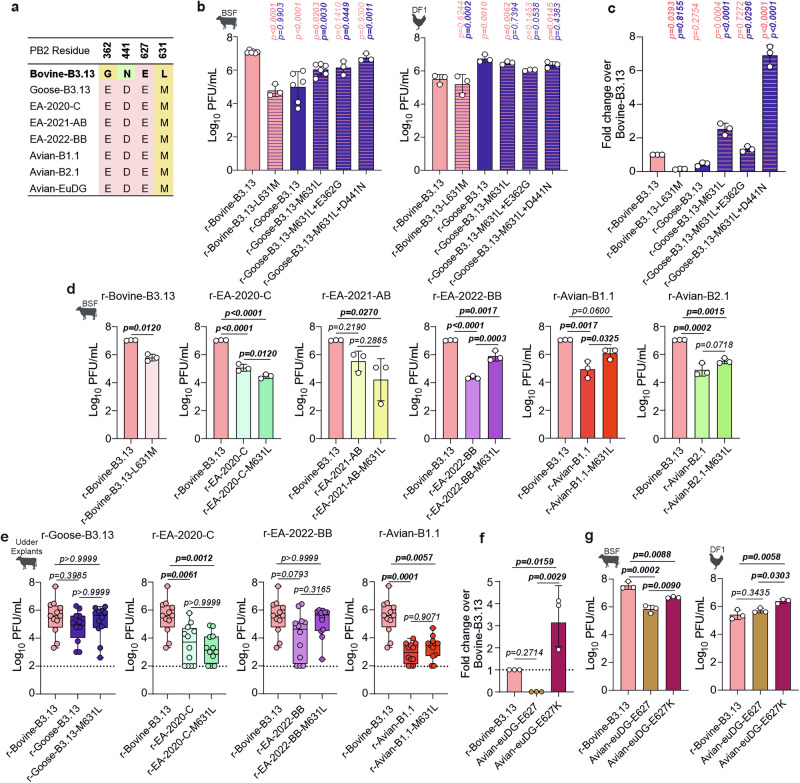
Replication of avian 2:6 viruses bearing M631L and other r-Bovine-B3.13 associated PB2 substitutions. **a** Alignment of PB2 residues against r-Bovine-B3.13 (bold). **b** BSF or DF1 cells infected with r-Bovine-B3.13 or r-Goose-B3.13 WT or mutant viruses carrying PB2 amino acid substitutions from the reciprocal virus. An MOI of 0.001 PFU/cell was used and virus in the supernatant at 24 hpi was titrated by plaque assay on MDCK cells. **c** Minireplicon assays in HEK293T cells at 24 hpt assessing the effects of PB2 amino acid substitutions in **b** on polymerase activity; data normalised to r-Bovine-B3.13. **d, e** Replication of WT or mutant r-Bovine-B3.13 and avian genotype representatives bearing PB2-M631L in BSF cells (**d**), or udder explants (**e**). **f** Minireplicon assays (as in **c**), comparing polymerase activity of r-Bovine-B3.13 and WT or PB2-E627K-bearing r-Avian-EuDG. **g** Replication of r-Bovine-B3.13 and WT or PB2-E627K-bearing r-Avian-EuDG in BSF and DF1 cells at 24 hpi (as in **b**). In **b–d** and **f–g**, data are mean +/- s.d. of three biological replicates (n = 3), except in **b** (n = 6 for r-bovine-B3.13, r-Goose-B3.13 and r-Goose-B3.13-M631L); Data were log-transformed and statistical differences between all groups were determined by one-way ANOVA with Tukey’s test (two-tailed; α = 0.05). In **e**, each data point represents one independently infected tissue explant (n = 12). Data were log-transformed and did not meet the assumption of normality. Box-and-whisker plots show the median (central line), interquartile range (box), and full data range (whiskers). Individual data points are overlaid. Statistical significance between virus groups was determined using the Kruskal–Wallis test with Dunn’s multiple comparisons (two-tailed; α = 0.05). P-values in bold indicate significance. (**b, d, e and g**) Created in BioRender. Bakshi, S. (2025) https://BioRender.com/732qna2.

Our data suggested that adaptive mutations in PB2 were important in defining phenotypic differences before- and after- emergence of B3.13 in cattle from an avian host. Importantly, only three amino acid residues differentiate the PB2 proteins of r-Goose-B3.13 and r-Bovine-B3.13 (E362G, D441N, M631L; Figs. 4i and 5a). Of these, the mutation PB2 M631L was previously shown to be an adaptive mutation in mammals^74,75^. Indeed, only a single 631 M mutation reduced the replication of r-Bovine-B3.13 to levels consistent with r-Goose-B3.13 in BSF cells (Fig. 5b). Introducing the PB2 M631L substitution did significantly enhance the replication of r-Goose-B3.13. However, it was not sufficient to fully recapitulate the phenotype conferred by the entire bovine PB2 as seen in Fig. 4j or enhance replication to levels observed for r-Bovine-B3.13 (Fig. 5b), suggesting that the additional substitutions, 362 G and 441 N, may further contribute to enhanced replication in bovine cells. Indeed, replication remained comparable to that of r-Bovine-B3.13 when either the E362G or D441N substitutions were individually introduced alongside M631L in the context of the Goose-B3.13 PB2 segment (Fig. 5b). Interestingly, the PB2 D441L + M631L substitution combination resulted in the greatest enhancement of replication, reaching titres within a 2-fold difference of the r-bovine-B3.13 virus, although this was not significantly different to the E362G + M631L combination. In polymerase assays (Fig. 5c), PB2 M631L alone boosted activity above that of the r-Bovine-B3.13 and r-Goose-B3.13 polymerases. Interestingly, the E362G + M631L combination boosted the r-Goose-B3.13 polymerase, but to levels below that of the single substitution. Conversely, the combination of D441N and M631L enhanced polymerase activity to levels far higher than that of the single substitution or parental polymerases. Together these results suggest that M631L plays an important role in bovine-adaptation, consistent with previous studies^75,80^, and further reveal that E362G and D441N provide an additive effect, enhancing replication beyond that of M631L alone in bovine cells.

All viruses tested in this study possess residue 631 M in PB2 except for r-Bovine-B3.13 (Fig. 5a). To further probe the role of PB2 M631L for avian virus replication in bovine cells, we introduced M631L into a selection of our 2:6 avian-origin recombinant viruses, including r-EA-2020-C, r-EA-2021-AB, r-EA-2022-BB, r-Avian-B1.1 and r-Avian-B2.1, and compared them to a r-Bovine-B3.13 (and a L631M reversion mutant) in bovine cells and in an ex vivo udder tissue system (Fig. 5d, e). L631M reduced r-Bovine-B3.13 replication in BSF cells, as expected (Fig. 5d). PB2-M631L enhanced replication of EA-2022-BB and r-Avian-B1.1 in BSF cells but did not significantly improve replication of r-EA-2020-C, r-EA-2021-AB, or r-Avian-B2.1, suggesting that the genomic context of this mammalian-adaptation is important. In ex vivo udder tissue, the replication pattern of M631L-viruses was comparable to that observed in BSF, although no significant differences were observed for any of the viruses tested (r-Goose-B3.13, r-EA-2020-C, r-EA-2022-BB, or r-Avian-B1.1; Fig. 5e). This is likely due to variability between explants in the ex vivo udder system, which makes it less suitable for detecting relatively small differences in replication efficiency (Fig. S3).

We also assessed additional adaptive PB2 mutations that have arisen in vivo in experimentally infected cows^66^. In a recent study, the PB2 E627K mutation was selected in vivo in experimentally inoculated dairy cattle infected with H5N1 2.3.4.4b of the euDG European genotype^66^. Without the E627K mutation, this virus grew comparably to avian virus genotypes that have been reported to successfully spillover into mammals, such as EA-2022-BB, B3.13 and D1.1 in BSF cells (Fig. 2b), suggesting the virus could replicate relatively efficiently within bovine cells without acquisition of the E627K substitution. We next compared the activity of the euDG polymerase with and without the E627K substitution. In human cells, polymerase activity was boosted significantly by this substitution, to levels far higher than the native euDG and Bovine-B3.13 polymerases (Fig. 5f). Next, we compared the 2:6 euDG virus against the PB2-627K bearing mutant, which exhibited increased replication in bovine cells (Fig. 5g), in line with previous observations that this 627K-bearing virus can replicate well in dairy cattle mammary tissue in vivo.

Next, we assessed whether additional mutations acquired by the B3.13 virus during its evolution in dairy cattle throughout the outbreak further enhanced replication in bovine cells. We introduced a series of substitutions in the internal genomic segments of our B3.13 Texas bovine virus (sampled 20^th^ March 2024) that accounted for changes that had been acquired later in the outbreaks in Colorado and California (after 3 and 5 months, respectively, of continuous circulation in cows) (Fig. S6). We observed no differences in the replication of WT or mutant r-Bovine-B3.13 viruses possessing PB2-E249G and NS1-R21Q (Colorado isolate) or PB2-K670R, PA-V432I and NS1-R67G (California isolate) in bovine cells at 24 h post infection.

### Sensitivity of 2.3.4.4b H5N1 to Mx1 and BTN3A3

**Fig. 6:**
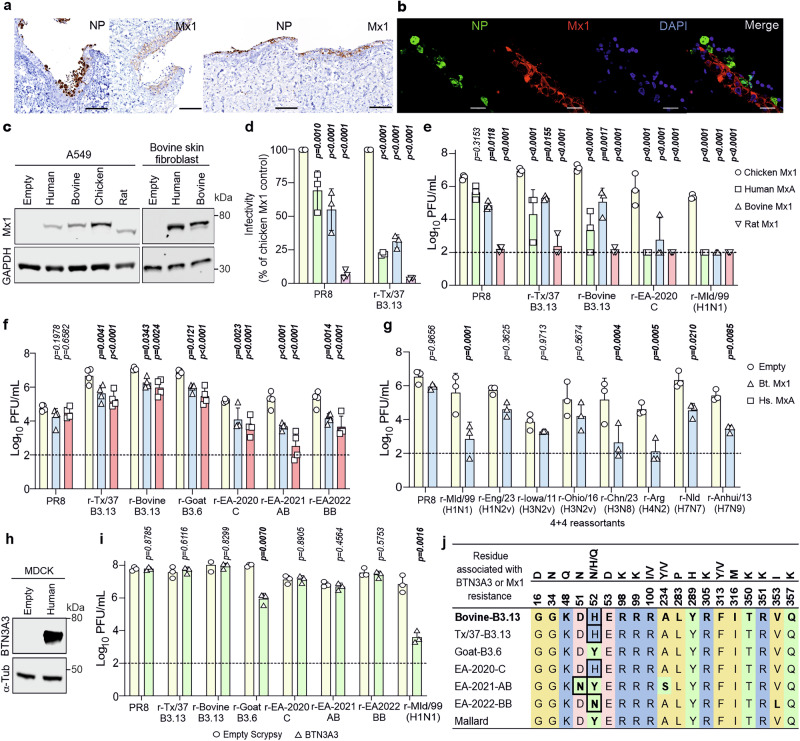
Effect of Mx1 and BTN3A3 on 2.3.4.4b reassortant viruses. **a** Identification of virus and Mx1 expressing cells by immunohistochemistry. Udder slices infected with r-Tx/37-B3.13. NP (brown) is visible in the epithelial layer lining the duct, while Mx1 is localised in the cytoplasm of ductal epithelial cells as shown by immunohistochemistry. Images are representative of experiments carried out in 8 different sections from two different animals. Scale bars, 100 μm. **b** Image showing detection of virus NP and Mx1 by immunofluorescence. NP and Mx1 show miminal colocalization in the duct epithelium. Scale bars, 20 μm. Immunofluorescence was performed on sections from the tissue in (**a**) as a final validation. **c**Western blot of lysates from A549 and BSF cells stably expressing human (MxA), bovine, chicken and rat Mx1. **d** Single-cycle infection of ZsGreen-expressing reporter viruses on modified A549 cells at 7 hpi. Titres normalised to Chicken Mx1 control cells. **e, f** A549 (**e**) or BSF (**f**) cells overexpressing Mx1 were infected at an MOI of 0.001 and infectious titres at 48 (**e**) or 24 (**f**) hpi were determined by plaque assay on MDCK cells. **g** As described in (**e, f**) except recombinant 4:4 viruses (Table S2) were used and peak titres of each virus (24 h or 48 h) are shown. **h** Western blot of lysates from MDCK cells overexpressing human BTN3A3. **i** Virus titres on modified MDCK cells overexpressing human BTN3A3 or transduced with an empty vector control. **j** Alignment of NP residues at positions associated with Mx1 or BTN3A3 resistance. Residues are aligned to r-Bovine-B3.13 (bold). Changes associated with resistance are boxed (bold). The data in (**d–f**) and (**g, i**) are mean +/- s.d. three biological repeats (n = 3), except for (**f**) where n = 4. Data in (**e–g and i**) were log-transformed and statistical significance was determined by two-way ANOVA with Tukey’s (**d–f**) or Šídák (**g, i**) multiple comparisons tests (two-tailed; α = 0.05). Only comparisons relative to untreated or chicken Mx1 controls are shown. P-values in bold indicate statistical significance. Gating data for (**d**) can be found in Fig. S7.

The host type-I/III interferon (IFN) response is an important barrier to virus cross-species transmission^55,60^. Upon infection, cells sense pathogen associated molecular patterns and initiate a signalling cascade resulting in the secretion of IFNs. These IFNs, in turn, induce an antiviral state in both infected and uninfected bystander cells, by stimulating the expression of hundreds of ISGs, some of which have direct antiviral activity and can inhibit virus replication, thereby limiting within-host spread in infected tissue, pathogenicity, and transmission^81–83^. We next assessed key ISGs known to preferentially restrict avian IAVs. MxA/Mx1 targets viral ribonucleoproteins (vRNPs)^55,57–60^ and restricts avian-origin viruses that do not possess escape mutations. A recent report showed that human MxA and bovine Mx1 are both effective at restricting a bovine B3.13 virus^84^. We expanded on this work by testing a broader diversity of IAVs in both human and bovine cells backgrounds. We confirmed that Mx1 is expressed in bovine udders following infection of precision cut bovine udder slices with r-Tx/37-B3.13 and examining Mx1 and NP staining by immunohistochemistry and immunofluorescence (Fig. 6a, b).

Mx1 acts on the viral NP^57^. As our 2:6 reassortants carry the NP of bovine influenza virus (and viruses of interest) they are a suitable model to evaluate the susceptibility of different IAVs to Mx1 restriction. To this end, we constitutively expressed human, bovine or rat Mx1 in bovine fibroblasts and human A549 cells (Fig. 6c). In human A549 cells, both bovine and human Mx1 displayed antiviral activity against an r-Tx/37-B3.13 reporter virus in a single-cycle infection relative to a chicken Mx1 control (lacking antiviral activity^85–87^) by approximately 3-fold, while PR8 was affected by less than two-fold relative to the chicken Mx1 control cells (Fig. 6d and Fig. S7). Neither bovine or human Mx1 proteins displayed the strength of antiviral activity exhibited by the rat Mx1 control, a known potent antiviral gene against IAV^88^. In a low MOI, multicycle assay, both human and bovine Mx1 exhibited antiviral activity against the B3.13 reassortants. However, the restriction of these viruses was less pronounced when compared to the avian viruses r-EA-2020-C or r-Mld/99(H1N1) (Fig. 6e). Furthermore, both human and bovine Mx1 proteins retained their antiviral properties against a wide array of both 2:6 and 4:4 reassortants when overexpressed in bovine skin fibroblasts (Fig. 6f–g) suggesting that these proteins can function in both the human and bovine cellular contexts. All reassortants based on 2.3.4.4b H5N1 were restricted by both bovine and human Mx1. As expected, all reassortants derived from avian viruses were inhibited by bovine Mx1 (Fig. 6f). In contrast, PR8 and reassortants originating from zoonotic swine viruses, r-Eng/23(H1N2v), r-Iowa/11(H3N2v) and r-Ohio/16(H3N2v), were not significantly restricted by bovine Mx1 (Fig. 6g). Hence, these results reinforce the notion that bovine Mx1 is an effective IAV restriction factor, as has been reported recently^84^, but also highlight that different IAVs vary in their susceptibility to this restriction.

Various residues in NP that dictate Mx1/MxA sensitivity/resistance have been reported previously^58,89–92^. Of the 2.3.4.4b recombinant viruses tested, only r-EA-2022-BB had any of the above resistance mutations (Y52N) (Fig. 6j), however this was not sufficient to overcome restriction by either human or bovine Mx1 proteins. Furthermore, the Y52H mutation, which has been described to help evade equine Mx1^92^, did not permit r-Bovine-B3.13 to evade human or bovine Mx1 proteins. This may suggest that either the viral context is important, or that bovine and human Mx1 proteins target different combinations of NP residues. From the more diverse panel of IAVs tested, the swine-origin human spillover viruses (r-Eng/23, r-Iowa/11 and r-Ohio/16) had all acquired R100I or R100V, and r-Eng/23 had also acquired F313V, and this might explain their relative resistance to bovine Mx1 (Fig. 6j).

Another ISG that specifically restricts most avian viruses, and also acts on the viral NP, is BTN3A3^55^. While Mx1^56–60^ is conserved in most vertebrates, including cattle, BTN3A3 originated specifically during the evolution of old world monkeys^55^, and therefore cannot play a role in H5N1 adaptation to cow cells. However, this restriction factor is a human genetic barrier to avian IAV zoonotic transmission. Hence, we assessed the replication of a selection of our reassortant viruses, including PR8 and r-Mld/99(H1N1) as controls, in MDCK cells overexpressing BTN3A3, and in the parental cells (Fig. 6h–i). As expected, PR8 replicated equally well in parental and BTN3A3 expressing cells, while r-Mld/99(H1N1) replication was substantially inhibited in the latter. Of the reassortant viruses tested, only r-Goat-B3.6 was restricted by BTN3A3, while all the other 2:6-based 2.3.4.4b or B3.13 viruses evaded this restriction factor (Fig. 6i). For BTN3A3, the residues involved in sensitivity/resistance described to date include sites 52 & 313 (with a combination of 52Y & 313 F/L being a sensitive genotype and one of 52H/N/Q and 313 V/Y being sufficient to provide resistance). The resistant phenotype, therefore, was not unexpected in the cases of r-Bovine-B3.13, r-Tx/37-B3.13, r-EA-2020-C, and r-EA-2022-BB, which each have either NP-52H or NP-52N^55^ (Fig. 6i, j). Likewise, r-Goat-B3.6 and r-Mld/99 both contained the sensitive genotype of NP-52Y and NP-313F^55^. Interestingly, however, r-EA-2021-AB was resistant to BTN3A3 activity, despite having NP-52Y & NP-313F, suggesting that other residue(s) are important. Hence, many 2.3.4.4b viruses already possess NP mutations that evade BTN3A3 restriction. We note that r-Goose-B3.13 was not tested in this series of experiments because its NP is identical to r-Bovine-B3.13.

### Increased modulation of the type-I IFN response by bovine-B3.13

**Fig. 7:**
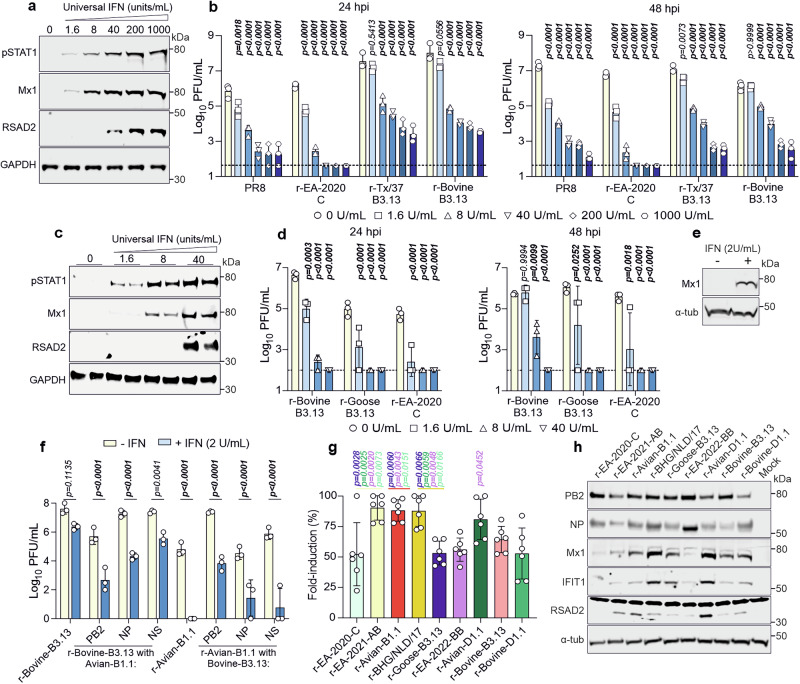
Sensitivity of 2.3.4.4b reassortant viruses to type I IFN in bovine cells. Immortalised BSF were pre-treated for 24 h with increasing concentrations of type I IFN prior to low MOI infection (0.001 PFU/cell). **a** Western blot of interferon-stimulated gene expression (pSTAT1, Mx1, RSAD2) at 24 h post IFN-treatment. **b** Virus titres in supernatants were determined by plaque assay on MDCK cells at indicated times post-infection. **c, d** As in (**a**) and (**b**) but including r-Goose-B3.13. **e, f** Replication of r-Bovine-B3.13, r-Avian-B1.1 and reciprocal segment swaps in the absence or presence of universal IFN. BSF were pre- or mock-treated with 2 U/mL IFN for 24 h prior to infection at MOI of 0.001. Western blot for Mx1 at 24 hours post-treatment (**e**), viral titres at 24 hpi were determined by plaque assay on MDCK cells (**f**)**. g**, Immortalised udder skin fibroblasts expressing an ISRE-firefly luciferase reporter infected with 2.3.4.4b 2:6 viruses at an MOI of 5 PFU/cell. Luminescence was measured at 24 hpi and normalised to mock controls. **h**, Western blot of viral proteins and ISGs in udder fibroblasts infected at an MOI of 5 for 24 hours. Data are mean +/- s.d. from three biological replicates (n = 3); each point is an average of technical duplicates. In **g**, n = 6, error bars show s.d. For **b, d and f**, data were log-transformed; statistical significances in **b, d and f** between IFN treated and untreated groups was assessed by two-way ANOVA with Dunnett’s (**b and d**) or Šídák (**f**) multiple comparisons tests (two-tailed; α = 0.05). For **g**, comparisons were determined by one-way ANOVA with Tukey’s test (two-tailed; α = 0.05). For **b, d and f**, P values in bold indicate statistical significance. For **g**, only significant values are shown.

Above we showed that bovine Mx1 exhibits antiviral properties and both avian and bovine-adapted viruses are restricted by this ISG. As highlighted above, expression of Mx1, and other antiviral ISGs, is under the control of the host the type-I/III IFN response, which is therefore in its complexity an important barrier to virus cross-species transmission^55,60^. We next compared the susceptibility of r-Bovine-B3.13, r-Tx/37-B3.13, and r-EA-2020-C to the bovine type-I IFN response. First, we confirmed that stimulation of bovine fibroblasts with universal type-I IFN resulted in the activation of markers of a type-I IFN response. As expected, western blot analysis of IFN-treated bovine fibroblasts showed a dose-dependent upregulation of phosphorylated Stat1 (pSTAT1), and of the ISGs Mx1 and RSAD2 (Fig. 7a). Next, we compared the susceptibility of r-Bovine-B3.13, r-Tx/37-B3.13, and r-EA-2020-C to IFN by assessing their replication in pre-stimulated or mock-treated bovine fibroblasts. All viruses showed sensitivity to IFN in a dose-dependent manner (Fig. 7b). However, r-EA-2020-C displayed a greater susceptibility to the bovine IFN response than the B3.13 viruses, or PR8 used as control. For example, at 24 hpi, pre-stimulation of bovine fibroblasts with 40U/ml of IFN was sufficient to completely inhibit replication of r-EA-2020-C below the detection limit, while both r-Bovine-B3.13 and r-Tx/37-B3.13 reached titres around 10^4^ PFU/ml in the same conditions. At 48 hpi, 1.6 U/ml of IFN had no effect on r-Bovine-B3.13 titres, but reduced r-EA-2020-C titres 150-fold on average (Fig. 7b, right). In addition, the avian B3.13-derived r-Goose-B3.13 was as similarly sensitive to IFN as r-EA-2020-C (Fig. 7c, d). To attempt to dissect this phenotype, we infected BSF pre-treated with 2 U/mL of IFN with some individual segment swaps between r-Bovine-B3.13 and r-Avian-B1.1, the latter representing one of the 2.3.4.4b genotypes in which PB2 M631L enhanced viral replication in bovine cells (Fig. 7e, f). We generated reassortants with segments 1, 5 and 8 (encoding PB2, NP and NS1/2) because B1.1 with bovine B3.13 PB2 provides an opportunity to assess an otherwise avian virus that replicates similarly to parental r-Bovine-B3.13 in the absence of IFN (Fig. 4g). In addition, NP is a target of Mx1 (and BTN3A3)^55–58^, and NS1 is a known viral modulator of the IFN response^93,94^. This experiment revealed that the parental r-Bovine-B3.13 was the least sensitive to 2 U/mL IFN treatment, as each of the avian B1.1 PB2, NP, and NS reassortants showed reduced virus replication in the face of an IFN response (Fig. 7f). Importantly, in the absence of IFN, r-Bovine-B3.13 with B1.1 NP or NS segment swaps and r-Avian-B1.1 with the Bovine-B3.13 PB2 replicated similarly to the parental r-Bovine-B3.13 virus. These data suggests that the bovine-B3.13 internal genes are better equipped to deal with an IFN antiviral state in bovine cells than the equivalent B1.1 counterpart genes, and highlighting a role for the PB2, NP and NS1 during bovine infection.

We next measured the ability of diverse recombinant 2:6 viruses to modulate the interferon response using primary bovine udder fibroblasts expressing a luciferase gene under the control of an interferon response element (ISRE) in bovine udder fibroblasts. We infected ISRE-luciferase reporter cells at MOI 5 PFU/cell and measured the induction of ISRE-driven luciferase. All viruses tested induced ISRE-dependent luciferase expression relative to mock-infected cells, although the fold-induction varied across the viruses tested. Viruses that were better at supressing ISRE-luciferase included r-EA-2020-C, r-Goose-B3.13, r-EA-2022-BB, r-Bovine-B3.13 and r-Bovine-D1.1 (Fig. 7g, h). In comparison, r-EA-2021-AB, r-AvianB1.1, r-BHG/NLD/17 and r-Avian-D1.1 were less efficient at supressing ISRE-luciferase expression. These data highlight the varied ability of genetically diverse H5N1 to supress ISG expression in infected bovine cells.

**Fig. 8:**
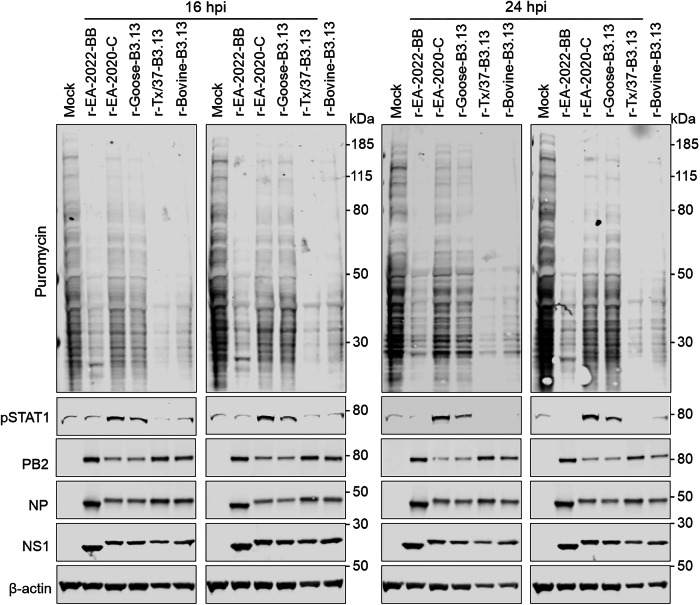
Host-shutoff activity of 2.3.4.4b reassortant viruses in bovine cells. Immortalised BSF were infected at an MOI of 5 PFU/cell for 16 h or 24 h and pulsed with puromycin for 1 hour. Cell lysates were assessed by western blot for total cellular proteins, phosphorylated STAT1 and the viral proteins PB2, NP and NS1 following puromycin-labelling. β-actin was assessed as a loading control. Western blot data are representative of two independent experiments.

To complement these experiments, we also tested a subset of viruses for their ability to induce host protein shutoff by measuring incorporation of puromycin into nascent proteins as a marker for efficient cellular protein synthesis. To this end, we infected BSF with a selection of reassortants at MOI 5 for 16 h or 24 h, then chased with puromycin for one hour before harvest. Our results (Fig. 8) shows that the ruminant-derived recombinant viruses (r-Bovine-B3.13 and r-Tx37-B3.13) and the avian r-EA-2022-BB induced a more severe cellular shutoff than r-EA-2020-C and r-Goose-B3.13, in line with their replication ability in these cells.

Collectively these results suggest that the ability to modulate the host IFN response also varies between 2.3.4.4b bovine-adapted and avian genotypes, with important differences, particularly among the latter.

## Discussion

H5N1 has been regarded as a global health threat since the emergence of the Gs/Gd lineage in poultry in 1996, with associated spillovers and fatal cases in humans. While numerous spillover events of avian IAV (including H5N1) from birds to humans and other mammals have been reported over the years prior to the most recent dairy cattle outbreaks, only a few avian viruses have established themselves as endemic mammalian lineages (Eurasian H1N1 in pigs^95^, H7N7 and H3N8 in horses^96–98^, and H3N2 in dogs^99^). The global emergence of the 2.3.4.4b clade has reignited justifiable concerns about the possibility of an H5N1 pandemic. Both the geographical distribution and the number of mammalian species infected by clade 2.3.4.4b viruses have dramatically increased compared to past H5N1 infection waves^7^. In addition, the current epizootic in dairy cattle provides the clearest example of a direct transfer of an entire avian IAV into a new mammalian host^100^. Importantly, mammal-to-mammal transmission of H5N1 2.3.4.4b has occurred also in other phylogenetically distant species such as marine mammals in South America^14,16,17,101^, and farmed fur animals in Europe^69,70^. While it is likely that the diversity of animal species affected by H5N1 reflects in part the increased geographical expansion of this virus^100^, we reasoned that the 2.3.4.4b clade may also possess intrinsic biological properties favouring spillover in mammals. Within the 2.3.4.4b clade, there are more than 80 genotypes determined by their internal gene cassette.

**Fig. 9:**
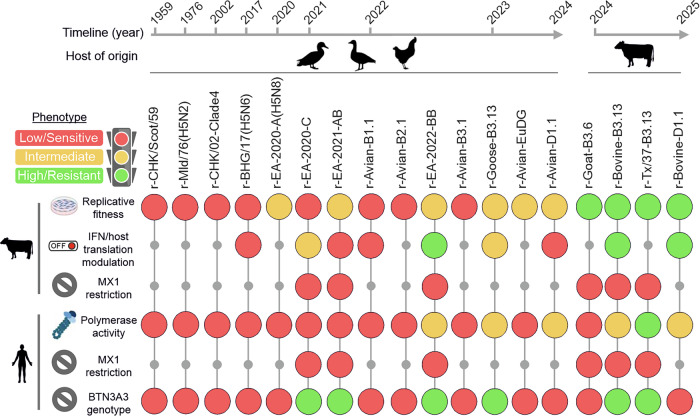
Phenotypes of the reassortant viruses observed in this study. Schematic diagram summarising the phenotypes of the reassortant viruses described in this study using either bovine or human cells and restriction factors as indicated. Note that summary of “IFN/host translational modulation” derives from results shown in Figs. 7 and 8. Created in BioRender. Bakshi, S. (2025) https://BioRender.com/90353mv.

Our study indicates that the constellation of internal genomic segments of the B3.13 and D1.1 viruses established in cows, and the B3.6 genotype that caused an outbreak in goats, provides a clear replication advantage in bovine cells over related avian 2.3.4.4b viruses and other IAVs originating from humans, pigs, horses and dogs. In addition, this phenotype is not confined only to cells from the udder, considering we tested additional bovine cell types. Hence, while HA-receptor preference and high virus titres in the milk and farming practices clearly favoured the spread of H5N1 in udders of dairy cows, the internal gene cassette of viruses such as B3.13 and D1.1 allows replication in more than one type of bovine cell. Importantly, we also show that the replicative fitness of avian H5N1 viruses in bovine cells varied greatly (data summarised in Fig. 9). Among the avian viruses, those with the highest replication efficiency in bovine cells included reassortants with internal gene cassettes belonging to genotypes that have spilled over in dairy cow, such as avian B3.13 and D1.1, in addition to those spilling over in fur farm mammals (EA-2022-BB), along with others including EA-2020-A, and euDG, that was shown to replicate in the udder of dairy cows experimentally infected via the intramammary route^66^. Other viruses, such as recombinants based on EA-2020-C, the North American B3.1, B2.1, B1.1, an early 2.3.4.4b virus isolated in 2017 in the Netherlands, and non 2.3.4.4b reached significantly lower titres in bovine cells. These observations highlight that multiple internal genomic constellations of avian H5Nx viruses may be ‘well suited’ to replicate in bovine cells and gives them an opportunity to subsequently acquire adaptive mutations and establish themselves in this new host. Conversely, other avian viruses appear poorly suited to replicate following a hypothetical spillover event.

Overall, our data suggest that H5N1 viruses possessing genetic traits conferring fitness in bovine cells are circulating in Europe and North America, and viral emergence might be impaired by other barriers such as agricultural practices. These results are in line with the successful experimental infection of dairy cow with an isolate representing the European avian H5N1 euDG genotype^66^. Indeed, the recent detection of H5N1 in a sheep in the UK provides a tangible example of the global risk that these viruses pose to ruminants regardless of whether the H5N1 outbreaks in North America are eventually controlled. The replication fitness in bovine cells may be a phenotype acquired with the evolution of the 2.3.4.4b clade. Indeed, our reassortants carrying the internal gene cassettes from H5N1 viruses isolated prior to the expansion of the 2.3.4.4b clade, including viruses isolated between 1959 to 2002, replicated particularly poorly. However, as summarised in Fig. 9, some avian-derived 2.3.4.4b viruses exhibited relatively poor replication efficiency in bovine cells, indicating there are some exceptions to this phenotype within the clade.

Using single and multiple segment swaps between recombinant viruses, our results suggest that the European avian 2.3.4.4b genotype (EA-2020-C) which likely seeded the original introduction to North America from Europe, requires multiple, simultaneous adaptive mutations in the polymerase complex and possibly NS segments, to replicate efficiently in bovine cells. Indeed, PB2, PB1, NP and NS segments were all required to switch the phenotype of the ancestral European r-EA-2020-C in bovine cells. Conversely, only the PB2 of a recombinant virus representing the closest sequenced American avian B3.13 predating the epizootic in cattle (r-Goose-B3.13), and for a distinct B1.1 genotype, was sufficient to switch the phenotype of r-Bovine-B3.13. Importantly, three non-synonymous mutations in PB2 differentiate r-Goose-B3.13 from r-Bovine-B3.13 (see below). Among these, the M631L mutation had been already shown to increase viral replication in mammalian cells^74,75,80^. Our data however suggest that the phenotype provided by this mutation is context dependent, as it does not enhance replication of all 2.3.4.4b viruses tested, and that in particular PB2 441 N may play an additive role in enhancing replication. Interestingly, this mutation arose independently in a ferret transmission study within a neuraminidase-inhibitor resistant variant of the H7N9 virus that caused extensive human spillovers^102^. Further, while this mutation was not widespread in avian B3.13 viruses closely related to the bovine-B3.13 virus^21^, it has been maintained in sub-clades of B3.13 bovine viruses (Fig. S6).

Of note, H5N1 A/Texas/37 (a B3.13 virus isolated from a human spillover case) acquired the well-described PB2-E627K mutation, a relatively frequent avian-to-mammalian mutation known to enhance interactions with human ANP32 proteins^48,103^. The E627K substitution is instead absent in the PB2 of both B3.13 and D1.1 bovine H5N1 (while the latter carries the well-known PB2 D701N, at least in virus causing the Nevada outbreak). Interestingly, using a panel of 33 distinct IAV polymerases in replicon assays, we showed that those carrying PB2 627K, including the human B3.13 H5N1 Tx/37, displayed the highest activity in human cells, significantly higher than the bovine- and goat-derived H5N1 polymerases. These are all polymerases derived from viruses that are either mammalian-adapted or derived from spillover events in humans. Our data in bovine cells using 4:4 reassortants with the polymerase complex of the viruses of interest, supported some of the differences noted above. Thus, overall, our data show clearly that adaptation of B3.13 and D1.1 (and B3.6), is bovine-specific and distinct from a broader polymerase adaptation to mammalian cells that could be detected in human cells (Fig. 9). Consistent with this notion, recombinant viruses possessing the internal genes of other mammalian-adapted viruses did not replicate as efficiently in bovine cells as those with bovine B3.13, D1.1 and goat B3.6 internal genes. Interestingly, in human cells there were little or no differences between the polymerase activity of the bovine-adapted H5N1 viruses and those of avian viruses from genotypes that have spilled over in bovine or fur animals (avian B3.13, D1.1 and EA-2022-BB). However, activities of this group of polymerases were higher than most other remaining avian polymerases. In mice experiments, r-Bovine-B3.13 caused more severe infection than avian r-EA-2020-C virus. We found the bovine and human B3.13 reassortant viruses were also less sensitive to the bovine type-I IFN response compared to avian EA-2020-C, an avian B3.13 ancestor, and an avian B1.1 virus. H5N1 B3.13 viruses were restricted by bovine (and human) Mx1, and human BTN3A3, two known restriction factors blocking avian IAV replication^56–60^. When we supplied an avian B1.1 virus with a bovine B3.13 PB2, so that replication in the absence of IFN was comparable to the bovine B3.13 equivalent, we observed a greater sensitivity to IFN in bovine cells. Similarly, when the bovine NP and NS segments were replaced with the avian B1.1 equivalent, this also increased IFN sensitivity, indicating adaptive roles for NP and NS in counteracting the IFN response. Generally, bovine-adapted viruses displayed a better ability to modulate IFN. Through puromycin assays, we show that reassortants carrying the internal genes of bovine-adapted B3.13 viruses induce stronger cellular shutdown, than avian B3.13 or EA-2020-C. Interestingly, EA-2022-BB induced a shutdown as strong as the bovine-adapted viruses. pStat1-inhibition, a marker of the IFN-response activation, was also directly correlated to the observed shutdown, and we obtained similar results in cells with a reporter gene under the control of the ISRE. We showed that the NP of bovine-adapted 2.3.4.4b viruses can escape human BTN3A3 restriction^55^, one of the human genetic barriers to avian IAVs. Thus, the spread of 2.3.4.4b viruses in several mammalian species, including its recent adaptation to cows, increases the pool of IAVs carrying genetic traits that counteract an important human barrier against zoonotic influenza, providing abundant ecological opportunities for spillover infections that might in turn facilitate adaption to humans^104^. For example, bovine Mx1 may provide a selective pressure on bovine H5N1 to select mutants that could increasingly escape both the bovine and human orthologues of this key restriction factor. These considerations, in addition to recent reports showing that a single amino acid residue in the viral HA is sufficient to switch its tropism from avian to mammalian receptors^43,44^, suggest that the zoonotic potential of H5N1 2.3.4.4b is relatively high. We acknowledge some limitations of our study. We specifically focused on the internal genomic segments of H5N1 viruses and used recombinant viruses harbouring the HA and NA of the laboratory-adapted PR8. Consequently, cells that may not be permissive to a recombinant virus harbouring the glycoproteins of PR8 may be susceptible to infection by an authentic 2.3.4.4b H5N1 virus and vice v*ersa*. For this reason, we used immortalised bovine skin fibroblasts and primary nasal fibroblasts as cell model systems. These cells allowed us to explore species-specific virus-host interactions within a bovine intracellular context as they express the required cellular co-factors, they are IFN-competent and express known IAV-restriction factors (e.g. Mx1). We selected key 2:6 viruses representing substantial IAV diversity and confirmed the phenotypes observed in these fibroblastic cells were recapitulated in ex vivo udder tissue from multiple animals. In addition, while reassortants are exceedingly useful tools to map molecular determinants of specific phenotypes, they may possess unforeseen defects due to incompatibilities between genome segments that have not co-evolved together. However, to mitigate for this risk, we always assessed the capacity of reassortants to replicate in chicken fibroblast cells (DF-1).

Overall, this study presents the first systematic evaluation of internal H5N1 gene segments from viruses representing 15 distinct H5Nx genotypes. We show that the H5Nx capacity to replicate in bovine cells varies throughout their evolutionary history, with earlier genotypes exhibiting reduced fitness. We show that although 2.3.4.4b viruses that spilled into cattle exhibit markedly enhanced replication and IFN evasion in bovine cells, many other avian-derived viruses from the same clade also possess internal gene constellations that support efficient replication in bovine cells and tissues, comparable to levels exhibited by those from other mammalian-adapted IAVs. For future risk assessment, we argue that characterising the molecular determinants of avian viruses showing intermediate replication phenotypes in bovine (or other mammalian) cells (Fig. 9) will be as crucial as identifying those mutations (e.g., M631L, E627K in PB2) that drive complete mammalian adaptation after spillover.

## Methods

### Biosafety considerations

All experiments with recombinant viruses were approved by the local genetic manipulation safety committee at the University of Glasgow (GM223), and the Health and Safety Executive of the United Kingdom and were carried out in appropriate laboratories by trained personnel at biosafety category level 2. All the 2:6 and 4:4 reassortant viruses used in this study were derived by reverse genetics using the HA and NA glycoproteins of the laboratory-attenuated vaccine strain A/Puerto Rico/8/1934 (H1N1)^105^, as done in previous studies^106,107^. Importantly, human challenge studies have shown PR8 to be highly attenuated or avirulent in human subjects^108,109^. Furthermore, the PR8 HA and NA cross-react with antibodies induced by the pH1N1 (pdm09-like) component of the seasonal influenza vaccine^110^, and PR8 NA is sensitive to the antiviral drugs oseltamivir and zanamivir^111,112^. The PR8 HA has a strong receptor preference for avian-type α2-3 linked sialic acid, further limiting tissue tropism of our recombinants^113^. No viruses were made with an HA gene containing a multi-basic cleavage site (a defining feature of highly pathogenic avian IAV). All the derived mutants used in this study contained engineered substitutions corresponding to those already present in circulating influenza A viruses. The 2:6 reassortants, generated by exchanging internal gene segments between 2:6 bovine-B3.13 and 2:6 avian viruses, were therefore not reasonably expected to replicate more efficiently than the parental 2:6 bovine-B3.13 virus, as the avian donor viruses exhibited significantly reduced replication in comparison. Work with reassortants used in this study was physically segregated from work with mammalian viruses with glycoproteins different from PR8 to avoid unintentional reassortant events. Overall, the benefits of understanding molecular determinants of cross-species spillover and host-adaptation, which can be used for risk assessment, surveillance strategies, and overall pandemic preparedness of circulating avian viruses, far outweigh the minimal, well-controlled experimental risks. This knowledge underpins global efforts to prevent future influenza pandemics.

### Ethical statement

All animal work was in accordance with the animal ethics and welfare committee at the University of Glasgow and the United Kingdom Home Office regulations (ASPA, 1986, PPL PP4085778). Work using animal tissues was approved by the Ethics Committee of the School of Veterinary Medicine of the University of Glasgow (ethics approval EA26/25). as well as the Ethics Committee of the Royal Veterinary College (Ethics Approval URN 2024 2312-2)

### Phylogenetic analysis

Influenza A virus sequences were retrieved from the NCBI Entrez databases using the Influenza A virus taxonomic ID with an in-house Python tool to access the E-utilities API. This dataset was further enriched with sequences from GISAID matching the search term “Clade=2.3.4.4b” and consensus genomes available in Andersen Lab’s public GitHub repository (https://github.com/andersen-lab/avian-influenza). All databases were last accessed on July 31^st^, 2025. Metadata associated with the sequences available on GISAID used in this study can be accessed via EPI_SET_250912fu (10.55876/gis8.250912fu). Fasta sequences were sorted by genomic segment and redundancies between databases were removed based on the name of the isolate. The MMseqs2 software tool v15.6f452 was used to cluster the sequences based on a 0.95 identity threshold and select a representative sequence per cluster (--cluster-mode 2 --cov-mode 1 --min-seq-id 0.95)^114^, resulting in the following number per genomic segment: PB2: 458, PB1: 406, PA: 405, NP: 221, MP: 112, NS: 222. Representative sequences per segment along with the sequences of the viruses of interest (detailed in Table S1) were aligned using MAFFT v7.453 using the default parameters^115^. Maximum likelihood trees were obtained with IQ-tree v2.1.2 under the best-fit model and plotted in RStudio using ggtree v3.12.0^116,117^.

### Generation of reassortant viruses harbouring PR8 HA and NA glycoproteins

pHW2000 reverse genetics plasmids used to rescue recombinant viruses based on A/California/04-061-MA/2009(H1N1) virus were a kind gift of Prof. Daniel Perez, the A/mallard/Netherlands/10-Cam/99(H1N1) virus from Prof. Laurence Tiley, A/Puerto Rico/8/34 (H1N1)^105^, and the A/canine/Illinois/11613/2015 (H3N2) from Prof. Luis Martinez-Sobrido. Each internal gene segment from A/equine/South Africa/4/2003(H3N8) was subcloned into pHW2000 following RT-PCR from RNA extracted from virus stocks. pHW2000 reverse genetics plasmids to rescue the remaining viruses described in Tables S1 and S2 were synthesised and cloned by GeneArt Thermo Fisher. pHW2000 plasmids to generate r-Goose-B3.13 were generated by site directed mutagenesis of r-Bovine-B3.13 by introducing in the latter non-synonymous mutations in PB2, PB1, PA, and NS1/NS2 to obtain genomic segment expressing viral proteins identical to A/goose/Colorado/2024, GISAID accession EPI_ISL_19228459.

2:6 viruses with the HA and NA genes from PR8 (Table S1) or 4:4 with the HA, NA, M and NS from PR8 (Table S2) were rescued by reverse genetics. HEK-293T (or cells modified with a lentiviral vector to overexpress *Gallus gallus* ANP32A; 293T-Gg.ANP32A. Annotated in Tables S1 and S2) cells were transfected with pHW2000 reverse genetics plasmids (250 ng of each plasmid in 6-well plate format) and the media changed to ‘virus growth medium’ (serum-free DMEM supplemented with 1 ug/mL TPCK-trypsin (Sigma) and 0.14% (w/v) BSA fraction V (Gibco) at 16 h post-transfection. Supernatant was collected at 48 h post-transfection and used to inoculate cultures of MDCK (or MDCK cells modified with a lentiviral vector to overexpress Gg.ANP32A; MDCK-Gg.ANP32A. Annotated in Tables S1 and S2) cells maintained in virus growth medium until approximately 70-80% of cells displayed cytopathic effect. At this point the supernatant was clarified by centrifugation prior to storage at –80 °C in small volume aliquots. All infections were carried out with previously unthawed virus aliquots to avoid freeze-thaw changes to infectious titre.

All viruses used were sequenced to confirm identity and identify spurious mutations. Virus stocks were homogenised in Trizol LS reagent (Thermo Fisher), and viral RNA was extracted using the Direct-zol-96 MagBead RNA kit (Zymo Research, R2102) automated on the KingFisher Flex System (Thermo Fisher) as per the manufacturers’ instructions. Extracted RNA was reverse transcribed and amplified in a single reaction using Superscript III one-step RT-PCR (Invitrogen) using primers specific for the conserved ends of the Influenza A genome segments^118^. Libraries were prepared with the SQK-NBC114.96 Oxford Sequencing Technologies (ONT) ligation sequencing kits with native barcoding and sequenced on a GridION sequencer using FLO-MIN114 flow cells. Oxford Nanopore reads were trimmed of adapters and checked for internal adapters using Porechop v0.2.4 (https://github.com/rrwick/Porechop). The cleaned reads were subsequently mapped to the reference sequence using minimap v2.22-r1101^119^. The consensus sequence was called using samtools consensus v1.21^120^ with settings showing all bases, inserts and deletions (arguments -a --show-ins yes --show-del yes). The consensus sequence was then compared to the reference sequence to verify that no mismatches were present.

### Cells

Cells were maintained in DMEM (Gibco 31966021) supplemented with 9% (v/v) HI-FCS (Gibco), 100 U/mL Penicillin and 100 µg/mL Streptomycin (Gibco) (hereafter referred to as ‘complete DMEM’) at 37 °C with 5% CO_2_ unless otherwise stated. HEK-293T, MDCK, A549 cells (and their Gg.ANP32A-overexpressing derivatives) have been described previously^55^. Primary-derived bovine skin fibroblasts have been described previously^121^. Nasal respiratory epithelium from the turbinates of the cow were collected from the abattoir in complete DMEM media containing 10% FCS, 100 U/mL Penicillin and 100 µg/mL Streptomycin (Gibco) and 2.5 µg/mL Amphotericin B as an antibacterial and antifungal agents respectively. Tissues were washed three times with PBS and incubated in 10 ml of complete DMEM media containing collagenase (2 mg/ml, collagenase from *Clostridium histolyticum*, Sigma) at 37 °C for 2 h. Subsequently, supernatant was strained with 70 μm strainer (Greiner) and subsequently centrifuged at 188 *x g* for 5 minutes. The cell pellet generated was washed three times in complete DMEM media to remove collagenase. Cells were finally cultured in complete DMEM and incubated at 37 °C, 5% CO_2_. Media was changed every day initially to remove non adherent cells until an adherent population of fibroblasts was achieved; cells were subsequently passaged using standard procedures. All infection experiments were carried out with early passages cells (before stationary phase was achieved) as described below.

Fibroblasts were extracted from udder tissue using a protocol like the one described above for the nasal fibroblasts. These cells were cultured in DMEM/F-12 (1:1) media (Gibco 11039021) supplemented with 9% (v/v) HI-FCS (Gibco), 100 U/mL Penicillin and 100 µg/mL Streptomycin (Gibco), 2.5 µg/mL Amphotericin B (Gibco) and 1X Insulin-Transferrin-Selenium-Ethanolamine (ITS -X) (Gibco) at 37 °C with 5% CO_2_ and were immortalized as previously described^122^. To generate an ISRE-luciferase reporter cell line, immortalized bovine udder fibroblasts were transduced with a lentiviral vector (pGreenFire1-ISRE Lentivector) kindly provided by Dr. Adam Fletcher. This construct drives the expression of GFP and firefly luciferase under the control of the human interferon response elements (ISRE) paired with a minimal CMV promoter.

BTN3A3 overexpressing cell lines have been described previously^55^. To overexpress Mx1, the *MX1* genes of *Rattus norvegicus* (GenBank accession NM_173096.3), *Bos taurus* (GenBank accession NM_173940.2), *Homo sapiens* (GenBank accession NM_002462.5) and *Gallus gallus* (GenBank accession NM_204609.2) were synthesised by BioBasic and subcloned into the pSCPRSY lentiviral vector. Cells were modified by lentiviral transduction using SCRPSY vectors that were generated by transfecting HEK-293T as described previously^123^. Cells were selected with puromycin (2 µg/mL, Gibco) and non-transduced cells were treated in parallel to validate selection.

### Virus replication assays

Typically, cells were plated in 12-well plates the day prior to infection. Cells were washed with serum-free DMEM prior to infecting with 200 µL of virus diluted in serum-free DMEM to achieve desired MOI (MOI stated in respective figure legends). After 1 h of incubation, the inoculum was removed, and cells were overlaid with 1 mL of virus growth medium. Plaque assays to titrate infectious virus in the supernatant of infected cells were performed in MDCK cells by inoculating cells washed with serum-free DMEM with a ten-fold serial dilution of sample (prepared in serum-free DMEM) and overlaying with virus growth medium (with 1.2% Avicel-RC591, IFF Pharma) after 1 h of adsorption.

### Quantification of viral RNA

RNA was extracted from infected culture supernatants homogenised in Trizol LS reagent as described above. To quantify negative-sense vRNA and avoid non-specific priming of positive-sense replication intermediates, quantification was carried out as a two-step RT-qPCR. Specifically, vRNAs were reverse transcribed using SuperScript IV (Thermo Fisher) and the Uni12 primer^124^ in half-reactions containing 2.5 ul of extracted RNA. qPCR was performed using Fast SYBR Green Master Mix (Thermo Fisher), and HA fragment specific primers: IAV_HA_For 5ʹ AAACAGCAGTCTCCCTTTCCAG and IAV_HA_Rev: 5ʹ GTTCCTTAGTCCTGTAACCATCCTCA, custom-synthesised by Merck (Sigma-Aldrich). Each reaction contained 4 ul of cDNA and was run using an ABI7500 Fast instrument, according to the manufacturer’s recommendations, and results were analysed with the 7500 Software v2.3 (Applied Biosystems, Life Technologies). A 714 bp synthetic gBlock carrying a partial sequence of PR8 HA (Twist Bioscience, custom) was used to generate a standard curve. Viral load was extrapolated from the curve and viral genomes were expressed as number of HA gene copies per ml of supernatant.

### Polymerase activity assays

Subconfluent monolayers of 293 T cells (1.5 ×10^5^ cells seeded in 24-well plates the day prior to transfection) were co-transfected with 10 ng each of pHW2000 bi-directional plasmids encoding PB2, PB1, PA and transfection control plasmid (CMV-driven expression of Renilla luciferase) and 25 ng each of NP and a PolI-driven expression of cRNA-sense Firefly luciferase reporter RNA, in Gibco™ Opti-MEM™ Reduced Serum Medium, using Invitrogen™ Lipofectamine™ 2000 CD Transfection Reagent. Transfection lacking the NP plasmid served as negative control. 24 hours post-transfection, the transfection medium was removed, and cells were lysed by adding 100 µl of 1X-passive lysis buffer (Dual-Luciferase reporter assay system, Promega), and freeze-thawing. The lysates were transferred to white-walled 96-well plates (Greiner) and luminescence was measured from 20 μL of lysate by using 30 μL of either luciferase assay reagent II or STOP & Glo reagent (Dual-Luciferase reporter assay system, Promega) in a GloMax® Navigator Microplate Luminometer (Promega) to measure IAV polymerase-driven Firefly luciferase activity and cellular-driven Renilla luciferase activity, respectively. Firefly luciferase values for every sample were normalised by their respective Renilla luciferase values to account for transfection efficiency variation between samples. Each sample was tested in duplicate in at least in three separate biological experiments.

### Mx1 and BTN3A3 susceptibility assays

For single-cycle titration of ZsGreen reporter viruses on A549-Mx1 cells, cells were cultured in 96-well plates 16 h prior to infection (1.5 ×10^4^ cells/well). Cells were washed with serum-free DMEM prior to infection with 100 µL of serum-free DMEM containing serially-diluted virus. At 7 h post-infection, cells were washed with PBS and dispersed with TrypLE (Gibco), resuspended in complete DMEM and fixed in 2% formaldehyde. To measure ZsGreen-positive cells, flow cytometry was performed on a Millipore GUAVA EasyCyte HT Flow Cytometer. Percentage of ZsGreen-positive cells was determined using FlowJo (https://www.flowjo.com) software (Fig. S3).

### Immunoblotting

Total cell lysates were prepared as previously described^125^. Lysates were heated at 75 °C for 10 min and subjected to polyacrylamide gel electrophoresis before being transferred to polyvinylidene difluoride (Merck Millipore IPFL00010) membranes at 30 V for 90 min. Membranes were blocked for 1 h with 1X Tris-buffered saline-0.2% Tween 20 (TBST) containing 5% dried skimmed milk, followed by incubation with primary antibodies diluted in 5% BSA/0.01% sodium azide in 1X TBST, overnight at 4 °C. After four 10-min 1X TBST washes, membranes were incubated with secondary antibodies (diluted in blocking buffer), for 1 h at room temperature protected from direct light. Following four more washes in 1X TBST, membranes were imaged using the LI-COR CLx-Odyssey Imaging platform. The primary antibodies used in this study are: PB2 - GeneTex (GTX125926), NP - MRC PPU Reagents and Services, Dundee (DA183, 5^th^ Bleed), NS1 – (MRC PPU Reagents and Services, Dundee (DA182, 2^nd^ Bleed), pSTAT1 (Tyr701) - Cell Signalling (9167S), IFIT1 - Origene (TA500948), RSAD2 - Proteintech (28089-1-AP), GAPDH - Cell Signalling (2118S), a-tubulin - Proteintech (66031-1-Ig), b-actin - Proteintech (66009-1-Ig), Mx1 - Proteintech (13750-1-AP) and puromycin (Millipore; MABE343). A Mx1 antibody raised in mice was kindly provided by Georg Kochs (University Medical Centre, Freiburg, Germany). Secondary antibodies used: Anti-rabbit IgG (H + L) (DyLight 800 4X PEG Conjugate) Cell Signalling (5151S), Anti-mouse IgG (H + L) (DyLight 680 Conjugate) Cell Signalling (5470S) and Anti-sheep IgG (H + L) (Alexa Flour^TM^ 680) Thermo Fisher Scientific (A21102).

### Precision-cut bovine udder slicing and in vitro culture

Precision-cut bovine udder slices (PCBUS) were prepared from udders of commercially slaughtered dairy cows without mastitis or any antibiotic treatment. Subsequently, a piece of approximately 10 cm x 10 cm x 5 cm was cut out of the middle region of the bovine udder, immediately transferred into Krebs-Henseleit solution and transported to the laboratory. Tissue was cut initially into slices of roughly 1 cm, and subsequently into tissue cores with a diameter of 8 mm. To stabilise the tissue in the tissue core holder, the tissue was embedded in 3% agarose (w/v in 0.9% NaCl). PCBUS were prepared with a Krumdieck tissue slicer with a thickness of 250-350 µm. The slicer was filled with ice-cold pre-oxygenated Krebs-Henseleit solution, adjusted to a pH of 7.4. Promptly, slices were placed in a 24-well plate in 1 mL of pre-warmed and pre-oxygenated medium. Incubating slices was done in RPMI-1640 medium supplemented with 1% penicillin-streptomycin, 1% fungizone and different concentrations of FCS (0%, 2% or 10%) at 38.5 °C in presence of 5% CO_2_. To assess cell viability, PCBUS were examined every 24 hours until the end of the experiment using AlamarBlue®. To evaluate cell integrity, HE-staining was performed. After infection, slices were fixed in 4% formalin at 4 °C for 24 h. Afterwards, slices were dehydrated in ethanol with increasing concentrations. Slices were subsequently cleared in xylene. Thereafter, slices were horizontally embedded in paraffin wax and sectioned at 2-3 μm. Prior to staining with haematoxylin and eosin (H&E), sections were deparaffinized and rehydrated. The cell viability of udder tissue was assessed by evaluating the cytoplasm and the shape/staining of nuclei as well as presence of necrosis and apoptosis.

### IFN-susceptibility assays

Immortalised bovine skin fibroblasts were seeded at a density of 2.5 ×10^5^ cells/well of a 24 well plate in complete DMEM media. Next day to immune prime the cells, media was removed and complete DMEM media containing indicated concentrations of Universal Type I IFN (Human IFN-Alpha Hybrid Protein from PBL Assay Science, Catalog Number: 11200) were added to the cells for 24 hrs. The next day, media was removed and cells were infected with indicated viruses at a MOI of 0.001 in serum free DMEM for 1 h at 37 C. After 1 h, virus was removed and virus overlay media (serum free DMEM, 0.14% fraction V BSA, 1x Penicillin/Streptomycin, 1ug/ml TPCK-treated trypsin) containing the same concentration of Universal IFN was added for continuous immune priming of cells. Virus in supernatant of infected cells at indicated timepoints was titrated by plaque assay on MDCK cells.

### ISRE-luciferase reporter assays

The ISRE-luciferase bovine udder fibroblasts were infected with r-EA-2000-C, r-EA-2021-AB, r-Avian-B1.1, r-BHG/NLD/17, r-Goose-B3.13, r-EA-2022-BB, r-Avian-D1.1, r-Bovine-B3.13 and r-Bovine-D1.1 at an MOI of 5 PFU/cell by spinoculation at 500 × *g* for 1 h at 12 °C. 24 hpi cells were lysed in 200 μl of 1X-passive lysis buffer (Dual-Luciferase reporter assay system, Promega). Post freeze-thawing, readings for firefly luciferase were obtained as mentioned previously for the polymerase assays.

### Protein shutoff assays

To examine protein shutoff, nascent proteins were labelled with puromycin as previously described^126^. Bovine skin fibroblasts were infected with r-Bovine-B3.13, r-Tx/37-B3.13, r-Goose-B3.13, r-EA-2020-C and r-EA-2022-B at a MOI of 5 PFU/cell by spinoculation at 500 × *g* for 1 h at 12 °C. 24 hpi, puromycin was added to the cells at a final concentration of 3.3 ng/mL for 1 h. Following cell lysis, samples were subjected to SDS-PAGE, and immunoblotting was performed for puromycin, viral PB2, NP & NS1 and b-actin. Quantification of puromycin was performed by normalising against levels of b-actin.

### Mouse infections

All mice were purchased from Charles River Laboratories (United Kingdom) and housed at the CRUK Scotland Institute on a 12 h light/dark cycle in rooms maintained at 20–24 °C and 45–65 % relative humidity. Mice (male, 8-week-old C57BL/6, *M. musculus*) were intranasally infected with 100 PFU of virus and monitored daily. At post-mortem, lungs were perfused and inflated with agarose and subsequently fixed in 8 % formalin for 24 h.

### Immunohistochemistry

Formalin-fixed and paraffin embedded (FFPE) mice lung and brain tissue blocks, or precision cut udder slices were cut into ~3 μm thick sections and mounted on glass slides for immunohistochemistry (IHC) as described previously^127^. The antibodies used were anti-Influenza A H1N1 Nucleoprotein (NP; A/WSN/1933; Novus Biologicals), anti-Cluster of Differentiation 3 (CD3; Agilent Dako), anti-Paired box protein-5 (Pax-5; Abcam). To detect Mx1, a custom-made monoclonal anti-Mx1 antibody was used (kindly provided by Georg Kochs and Hartmut Hengel, Universitaetsklinikum Freiburg, Germany). As a negative control, the primary antibody was replaced by Dako Real Antibody Diluent (Agilent Dako; Code S2022). Visualisation was performed using the Dako EnVision+ System-HRP Labelled Polymer Anti-Rabbit (Agilent Dako; code K4001) in an automated stainer (Dako Autostainer Link 48, Agilent Technologies). 3,3’-Diaminobenzidine (DAB) was used as a chromogen in all IHC experiments. For confocal imaging, the same primary antibodies (with the same concentrations) have been used as above. Secondary antibodies included AlexaFluor-488 and -594 (codes A11034, A21203; Thermo Fisher Scientific). Dapi was used for nuclear staining. Images have been captured with a Zeiss LSM 710 Confocal Microscope (Zeiss, Wetzlar, Germany).

### Digital pathology

Slides were digitised and scanned at 20x magnification using the Aperio Versa 8 Slidescanner and Aperio Versa 1.0.4.125 software (Leica Biosystems) for subsequent image analysis. Images of digitised slides were captured using the Aperio ImageScope software v12.4.3.5008 (Leica Biosystems). Positive cell detection and quantification of CD3 and Pax-5, as well as positive pixel quantification of NP, were performed using QuPath software (version 0.4.3;^128^) with settings adjusted to optimise signal detection for each marker^127^. To create the thresholder, the ‘Resolution’ was set to 1.09 μm/pixel or higher. For the ‘Channel’, we used ‘DAB’ (IHC); the ‘Prefilter’ was always ‘Gaussian’ while ‘Smoothing sigma’ as well as the ‘Threshold’ have been tuned for each set of experiments to optimise the correct detection. The ‘Above threshold’ option was always set to ‘Positive’ whereas the ‘Below threshold’ was always set to ‘Negative’. The ‘Region’ to be analysed was set to ‘Any annotation ROI’. The readout is a percentage of positive pixels per total pixels in the annotated area.

The ‘Positive cell’ detection feature has been used instead for the detection of cell membrane-associated CD3-positive cells and nucleus-localised Pax-5. The settings for the ‘Requested pixel size’ were adjusted to 0.5 μm or smaller and the ‘Nucleus parameters’ included a ‘Background radius’ of 8 μm. The ‘Median filter radius’ was set to 0.8 μm or smaller, ‘Sigma’ to 1.5 μm or smaller while the ‘Minimum’ and ‘Maximum’ areas were adjusted to 3–5 μm^2^ and 70-200 μm^2^ respectively. The ‘Intensity parameters’ included a ‘Threshold’ of 0.1 and a ‘Max. background intensity’ of 2. The ‘Cell parameters’ included a ‘Cell expansion’ of 3-4.4286 μm. The readout is a percentage of positive cells detected per all cells in the annotated area.

### Statistical analysis

Statistical analyses were performed using GraphPad Prism v10. Data were log10-transformed or normalised as described in the figure legends. Normality was assessed using the Shapiro-Wilk test. Statistical tests were applied as described in the figure legends. All statistical tests were two-tailed. Non-parametric tests were used for non-normal datasets where appropriate. Statistical significance was defined as P  <  0.05.

### Figure preparation

Figures were assembled using CorelDRAW (2025) and schematic elements (e.g. silhouettes), were created with BioRender (https://biorender.com) and Phylopic^129^.

### Software

Western blotting data were collected on an Odyssey CLx infra-red imaging system (LICOR CLX-2789). qPCR assays were performed on an Applied Biosystems™ 7500 Fast Real-Time PCR machine. Guava InCyte 3.3 (https://www.merckmillipore.com/GB/en/20130828_204624?ReferrerURL=https%3A%2F%2Fwww.google.com%2F&bd=1) was used to acquire flow cytometry data.

Influenza A virus sequences were retrieved from the NCBI Entrez databases using the Influenza A virus taxonomic ID with an in-house Python tool to access the E-utilities API. Tissue slides were digitised and scanned at 20x magnification using the Aperio Versa 8 Slidescanner and Aperio Versa 1.0.4.125 software (Leica Biosystems). Confocal microscopy images were captured with a Zeiss LSM 710 Confocal Microscope (Zeiss, Wetzlar, Germany). Luciferase activity was collected on a Promega GloMax Luminometer. GraphPad Prism 10 (https://www.graphpad.com/scientific-software/prism/) was used for plotting graphs and statistical analyses. qPCR data was analysed on Applied Biosystems QuantStudio Design & Analysis Software 2.6.0. FlowJo (https://www.flowjo.com) was used to analyze flow cytometry data using the most up-to-date software available. Western blotting data were analyzed on Image Studio Lite https://www.licor.com/bio/image-studio-lite/). To analyse Sanger sequencing data, DNA Dynamo software (https://www.bluetractorsoftware.com) was used. The MMseqs2 software tool v15.6f452 was used to cluster sequences as described fully in Materials and Methods. MAFFT v7.453 was used to align sequences as described in Materials and Methods. Maximum likelihood trees were obtained with IQ-tree v2.1.2 under the best-fit model and plotted in RStudio using ggtree v3.12.0. Images of digitised slides were captured using the Aperio ImageScope software v12.4.3.5008 (Leica Biosystems). Positive cell detection and quantification of CD3 and Pax-5, as well as positive pixel quantification of NP, were performed using QuPath software (version 0.4.3).

### Reporting summary

Further information on research design is available in the Nature Portfolio Reporting Summary linked to this article.

## Supplementary information

Supplementary Information

Description Of Additional Supplementary File

Supplementary data 1

Reporting summary

Transparent Peer Review file

## Source data

Source Data

## Data Availability

The underlying data generated in this study have been deposited in the University of Glasgow Enlighten open access database under accession code 10.5525.gla.researchdata.2087 (10.5525/gla.researchdata.2087)^130^. Source data are provided with this paper.
