# Supplementary material for: The potential of H5N1 viruses to adapt to bovine cells varies throughout evolution
Source: Nat Commun. 2025 Dec 15;16:11042. doi: 10.1038/s41467-025-67234-1 (PMC12706089; doi:10.1038/s41467-025-67234-1)
Supplement: Supplementary file 1 — Supplementary Information [file 41467_2025_67234_MOESM1_ESM.pdf]

**Table S1 – 2:6 Reassortants used in this study.** Each virus has the HA and NA segments from PR8, and all remaining segments from the virus listed

| Abbreviated name | Internal genes origin | Sequence details | Non-synonymous mutations detected in viral stocks |
|---|---|---|---|
| PR8 | A/Puerto Rico/8/1934 (H1N1) | GenBank EF467817 to EF467824 | None |
| r-Bovine-B3.13 | A/dairy cow/Texas/24-008749-001-original/2024 (H5N1) | GISAID EPI_ISL_19014384 | None |
| r-Tx/37-B3.13 | Human A/Texas/37/2024 (H5N1) | GISAID EPI_ISL_19027114 | None |
| r-Bovine-D1.1 | A/dairy cow/Nevada/002645-005/2025 | GISAID EPI_ISL_19716907 | None |
| r-Goat-B3.6 | A/goat/Minnesota/24-007234-003-original/2024 (H5N1) | GISAID EPI_ISL_19015123 | None |
| r-EA-2020-C | A/chicken/England/053052/2021 (H5N1) | GISAID EPI_ISL_9012457 | None * |
| r-EA-2021-AB** | A/chicken/Scotland/054477/2021 (H5N1) | GISAID EPI_ISL_9012696 | PA D347Y |
| r-EA-2022-BB | A/chicken/England/085598/2022 (H5N1) | GISAID EPI_ISL_13782459 | None |
| r-Goose-B3.13 | r-Bovine-B3.13 with non-synonymous mutations to represent A/goose/Colorado/2023 | GISAID accession EPI_ISL_19228459 | None |
| r-Avian-euDG | A/wild goose/Nordrhein-Westfalen/2024AI00581/2024 (H5N1) | GISAID EPI_ISL_19353476 | None |
| r-BHG/17 | A/Black-headed Gull/Netherlands/29/2017 | EPI_ISL_289714 | None |
| r-EA-2020-A(H5N8) | A/duck/Chelyabinsk/1207-1/2020 (H5N8) | GISAID EPI_ISL_637098 | None |
| r-Avian-B1.1 | A/Baikal Teal/New York/USDA-009119-001/2022 (H5N1) | GISAID EPI_ISL_18132961 | NA Q258R |
| r-Avian-B2.1 | A/American Crow/Minnesota/USDA-012775-001/2022 (H5N1) | GISAID EPI_ISL_18133061 | NA Q258R |
| r-Avian-B3.1 | A/American_White_Pelican/SK/FAV-0912-12/2022 (H5N1) | GISAID EPI_ISL_19154047 | None |
| r-Avian-D1.1 | A/Red_Tailed_Hawk/BC/AIVPHL-2513/2024 (H5N1) | GISAID EPI_ISL_19533305 | None |
| r-CHK/Scot/59*** | A/chicken/Scotland/1959(H5N1) | GenBank: GU052525.1, GU052524.1, GU052523.1, CY015084.1, CY015082.1, CY015085.1 | None |
| r-Mld/76(H5N2)*** | A/mallard duck/ALB/57/1976 (H5N2) | EPI_ISL_8774 | None |
| r-HK/CHK/02-Clade4 | A/chicken/Hong Kong/409.1/2002 (H5N1) | GISAID EPI_ISL_67724 | None |
| r-pdm09(H1N1) | A/California/04-061-MA/2009(H1N1) | GenBank KX134889 (PB2), MH393723.1 (PB1), MH393724.1 (PA), MH393698.1 (NP), KX136570 (M), NS: KX134783 (NS). | PA E298K, NP D101G |
| r-Eng/23(H1N2v) | A/England/2023 (H1N2v) | GISAID EPI_ISL_18548251 | None |

| | | | |
|---|---|---|---|
| r-Canine(H3N2) | A/canine/Illinois/11613/2015 (H3N2) | doi:10.1371/journal.ppat.1008409 | PA C489S |
| r-Equine(H3N8) | A/equine/South Africa/4/2003(H3N8) | GenBank ON797667 to ON797674 | PA V51A & PA C478S |

* PB2 D701A mutation was detected in one of five independently rescued stocks used in this study (data described in Fig S5b) and this did not alter the virus titre in bovine skin fibroblasts.

** PB2 D701N mutation was detected in the r-EA-2021-AB bearing the M631L substitution, but this virus grew to lower titres than the WT r-EA-2021-AB virus.

*** Viruses indicated were rescued in 293T-Gg.ANP32A and propagated on MDCK-Gg.ANP32A cells due to failure to rescue in respective parental cells.

**Table S2 – 4:4 Reassortants used in this study.** Each virus has the HA, NA, M and NS segments from PR8, and all remaining segments from the virus listed

| Abbreviated name | Internal genes origin | Sequence details | Non-synonymous mutations detected in viral stocks |
|---|---|---|---|
| r-Mld/99(H1N1) | A/mallard/Netherlands/10-Cam/1999(H1N1) | GenBank KC209512 to KC209519 | PA C489S |
| r-Bovine-B3.13* | A/dairy cow/Texas/24-008749-001-original/2024 (H5N1) | GISAID EPI_ISL_19014384 | None |
| r-Tx/37-B3.13* | Human A/Texas/37/2024 (H5N1) | GISAID EPI_ISL_19027114 | None |
| r-Goat-B3.6* | A/goat/Minnesota/24-007234-003-original/2024 (H5N1) | GISAID EPI_ISL_19015123 | None |
| r-EA-2020-C* | A/chicken/England/053052/2021 (H5N1) | GISAID EPI_ISL_9012457 | None |
| r-EA-2021-AB* | A/chicken/Scotland/054477/2021 (H5N1) | GISAID EPI_ISL_9012696 | None |
| r-EA-2022-BB* | A/chicken/England/085598/2022 (H5N1) | GISAID EPI_ISL_13782459 | None |
| r-Goose-B3.13 | r-Bovine-B3.13 with non-synonymous mutations to represent A/goose/Colorado/2024 | GISAID accession EPI_ISL_19228459 | None |
| r-Eng/23(H1N2v) | A/England/2023 (H1N2v) | GISAID EPI_ISL_18548251 | None |
| r-Anhui/13(H7N9) | A/Anhui/2013 (H7N9) | GISAID EPI_ISL_17264823 | None |
| r-Iowa/11(H3N2v) | A/Iowa/08/2011 | EPI_ISL_99214 | PB1 D638V |
| r-Ohio/16(H3N2v) | A/Ohio/27/2016 | OH/16: EPI_ISL_232044 | None |
| r-Chn/23(H3N8) | A/Guangdong/ZS-2023SF005/2023 | EPI_ISL_17464053 | None |
| r-Arg(H4N2) | A/silver teal/Argentina/CIP051-32/2011 | OQ821651.1, OQ821652.1, OQ821653.1, OQ821655.1 | None |
| r-Nld(H7N7) | A/Netherlands/219/03(H7N7) | AY342413.1, AY340083.1, AY342418.1, AY342425.1 | None |

*Viruses indicated were rescued in 293T-Gg.ANP32A and propagated on MDCK-Gg.ANP32A cells due to failure to rescue in respective parental cells without incurring known mammalian-adaptive mutations

**Table S3 – Viruses tested exclusively in polymerase activity assays in this study.** The polymerase of these viruses was tested in polymerase activity assays described in Fig 3.

| Abbreviated name | Full virus name | Sequence details |
|---|---|---|
| Hkg/68 (H3N2) | A/Hong Kong/01/1968(H3N2) | GenBank: PB2 KY321924.1, PB1 KY321925.1, PA KY321926.1 and NP KY321928.1 |
| Chn/13 (H10N8) | Using A/Jiangxi-Donghu/346/2013 (H10N8) | GenBank: PB2 MK070525.1, PB1 PA MK070526.1, NP MK070527.1, NS MK070529.1 |
| Nor/18 (H3N2) | A/Norway/3275/2018 (H3N2) | EPI_ISL_390020 |
| Anhui/13 (H7N9) | A/Anhui/1/2013 (H7N9) | GISAID EPI_ISL_17264823 |
| Nor/18 (H1N1) | A/Norway/3433/2018 (H1N1) | EPI_ISL_391292 |

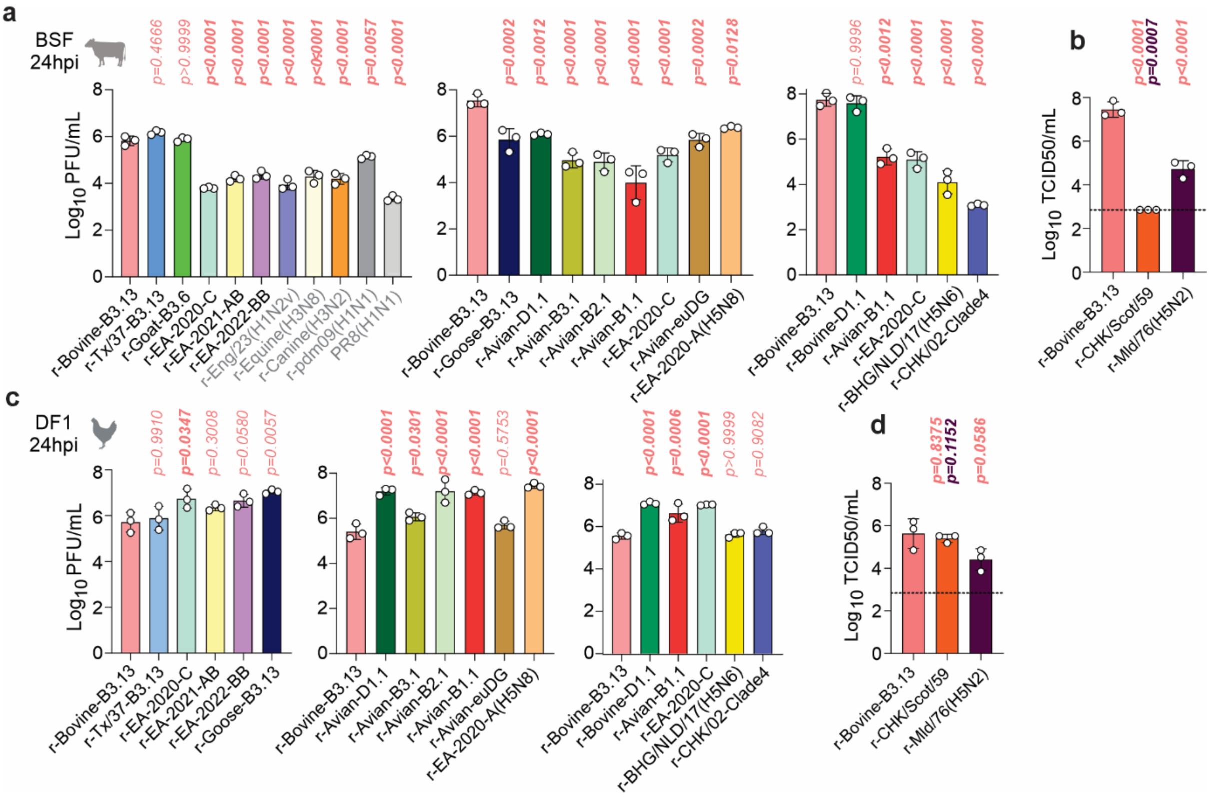


**Fig S1. Replication of 2.3.4.4b and other IAV reassortant viruses in bovine skin fibroblasts (BSF). a**, BSF were infected with 0.001 PFU per cell and infectious virus in the s upernatant at 24 hours post infection (hpi) was quantified by plaque assay on MDCK cells, or by TCID50 on DF-1 cells **b**. **c-d**, Same as above (a-b), but chicken embryonic fibroblast (DF1 ) cells were infected instead. Data are mean +/- s.d. of three independent biological exp eriments (n = 3). Data were log-transformed and confirmed to be normally distributed using t he Shapiro-Wilk Test. Multiple comparisons between all reassortants were performed using a n ordinary one-way ANOVA with Tukey's multiple comparison test (two-tailed; α= 0.05), although only comparisons relative to controls are shown. P-values in bold indicate statistical significance. H5N1 viruses are shown in black, while labels for non-H5N1 IAVs are shown in grey. (**a and c**) Created in BioRender. Bakshi, S. (2025) https://BioRender.com/duevd5g

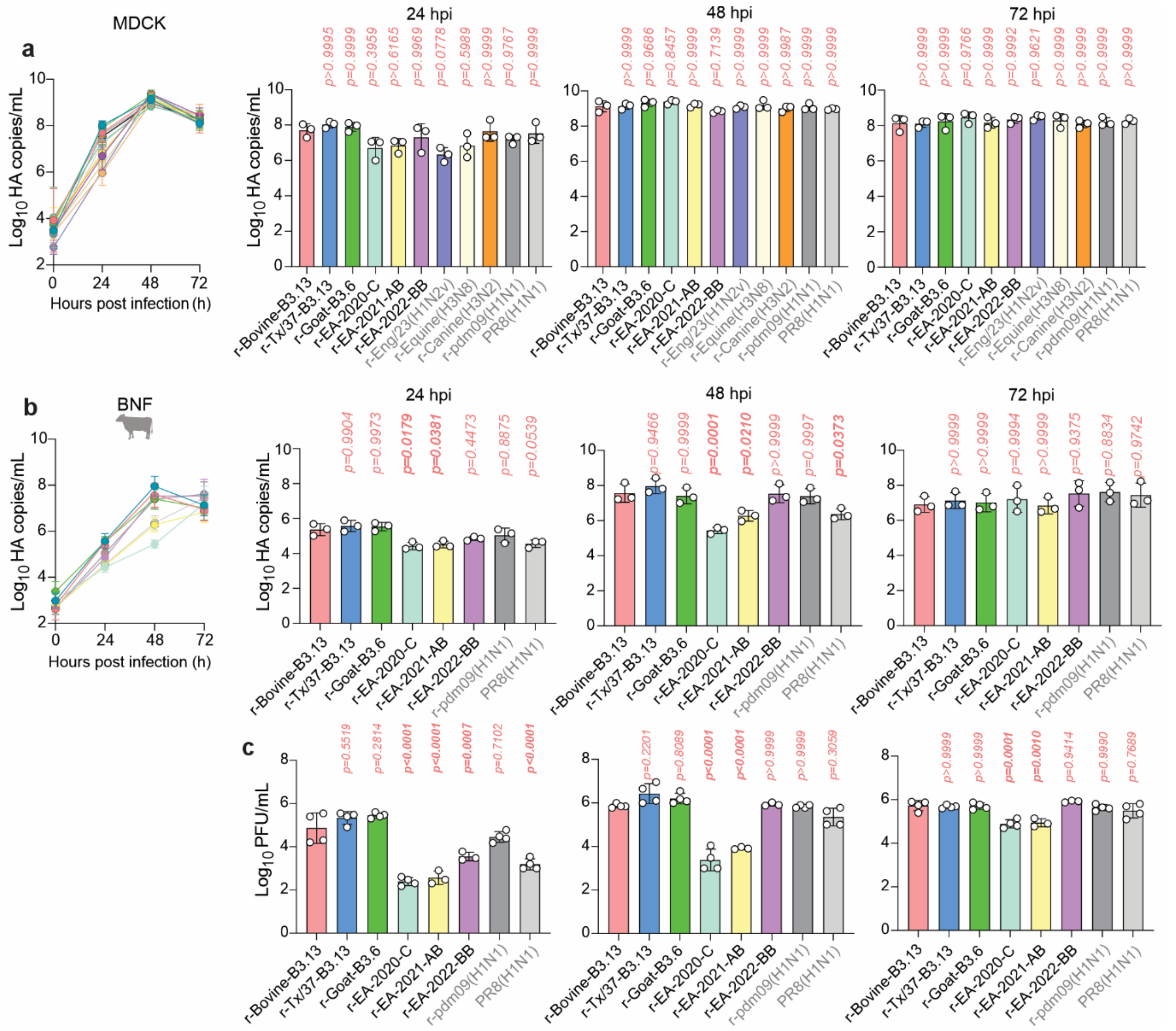


**Fig S2. Replication of 2.3.4.4b and other IAV reassortant viruses.** **a**, MDCK cells were infected with 0.0005 genome copies per cell and genomic copies in the supernatant at indicated times post infection were titrated by RT-qPCR. **b-c**, Bovine nasal fibroblasts (BNF) cells were infected as in **a** and viral genome copies or infectious virus in the supernatant were titrated by RT-qPCR (**b**) or plaque assay on MDCK cells (**c**). Data are mean +/- s.d. of three (n = 3) or four (n = 4) biologically independent experiments in **a-b** and **c**, respectively. For RT-qPCR experiments, individual points represent the mean of technical duplicates for each biological repeat. Data were log-transformed and confirmed to be normally distributed using the Shapiro-Wilk Test. Multiple comparisons between all groups were performed using an ordinary one-way ANOVA with Tukey's multiple comparison test (two-tailed; α= 0.05), although only comparisons relative to controls are shown. P-values in bold indicate statistical significance. H5N1 viruses are shown in black, while labels for non-H5N1 IAVs are shown in grey. (**b**) Created in BioRender. Bakshi, S. (2025) https://BioRender.com/jvfayku

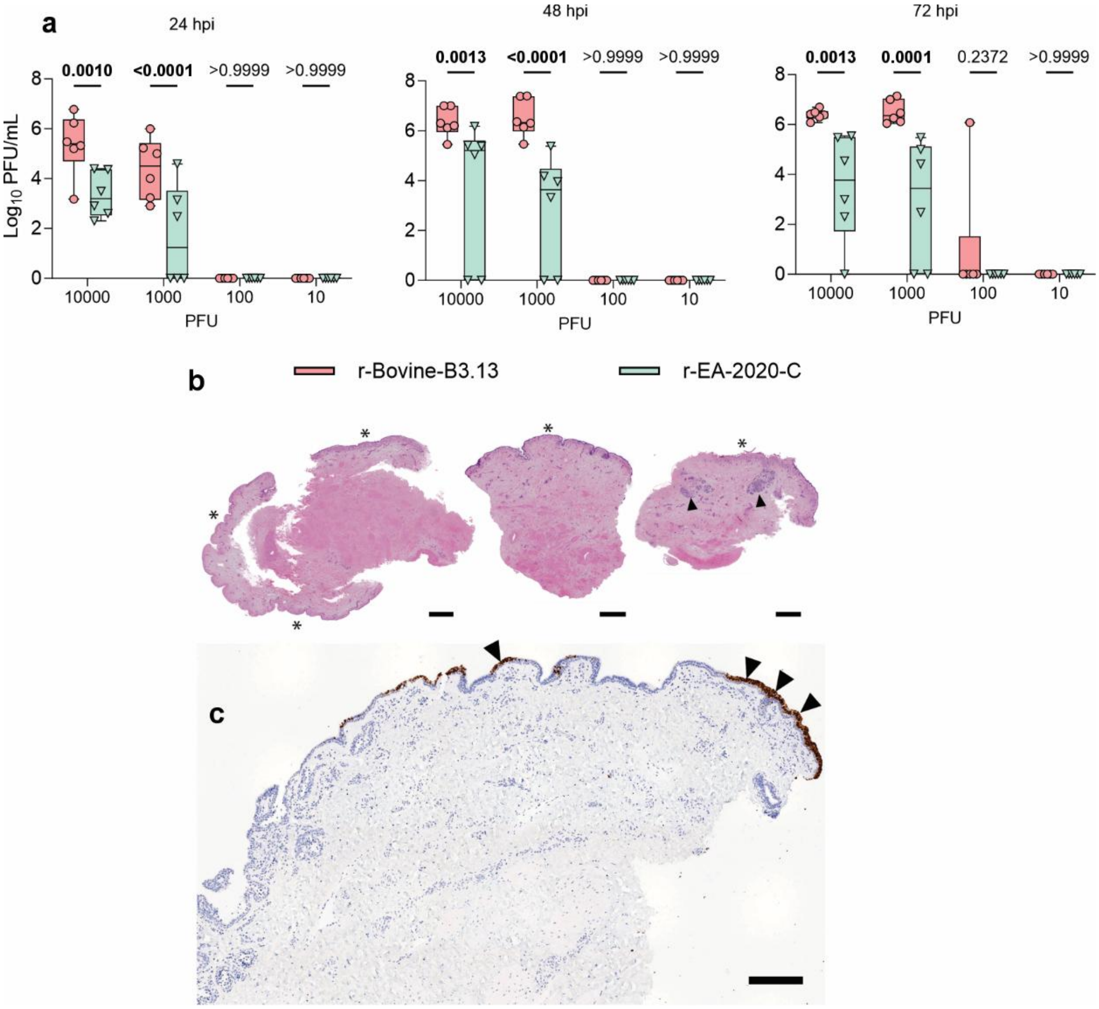


**Fig S3. Optimization of Infection of Bovine udder tissue explants**. **a**, Udder tissue explants were infected with 10, 100, 1000 or 10000 PFU per explant and infected for indicated time points. Infectious virus in the supernatant was quantified by plaque assay on MDCK cells. Data are from six independently infected tissue explants from three biological donors (n = 6). Data were log-transformed and statistical significance between virus groups was determined using a two-way ANOVA with Tukey's multiple comparison (two-tailed; α= 0.05). Boxes represent the interquartile range, the lines indicate the median, and whiskers show the full data range. P-values in bold indicate significance. **b**, Samples show high variability in size and shape: larger sample with larger areas of epithelium surrounding the sample (*, left, infected with r-EA-2022-BB); smaller samples with epithelium on the top only (*, middle, infected with r-Bovine-B3.13); and samples with epithelium on the top (*) and subepithelial glands (arrow heads, right, infected with r-EA-2020-C). **c**, Immunohistochemistry of explants detecting NP in the cytoplasm of cells in the epithelium (arrow heads) next to epithelial areas which are negative. HE staining in **b**, bars in **b** =500 μm. Bar in **c**, 200 μm. Images shown in (**b-c**) are derived from three explants in one experiment.

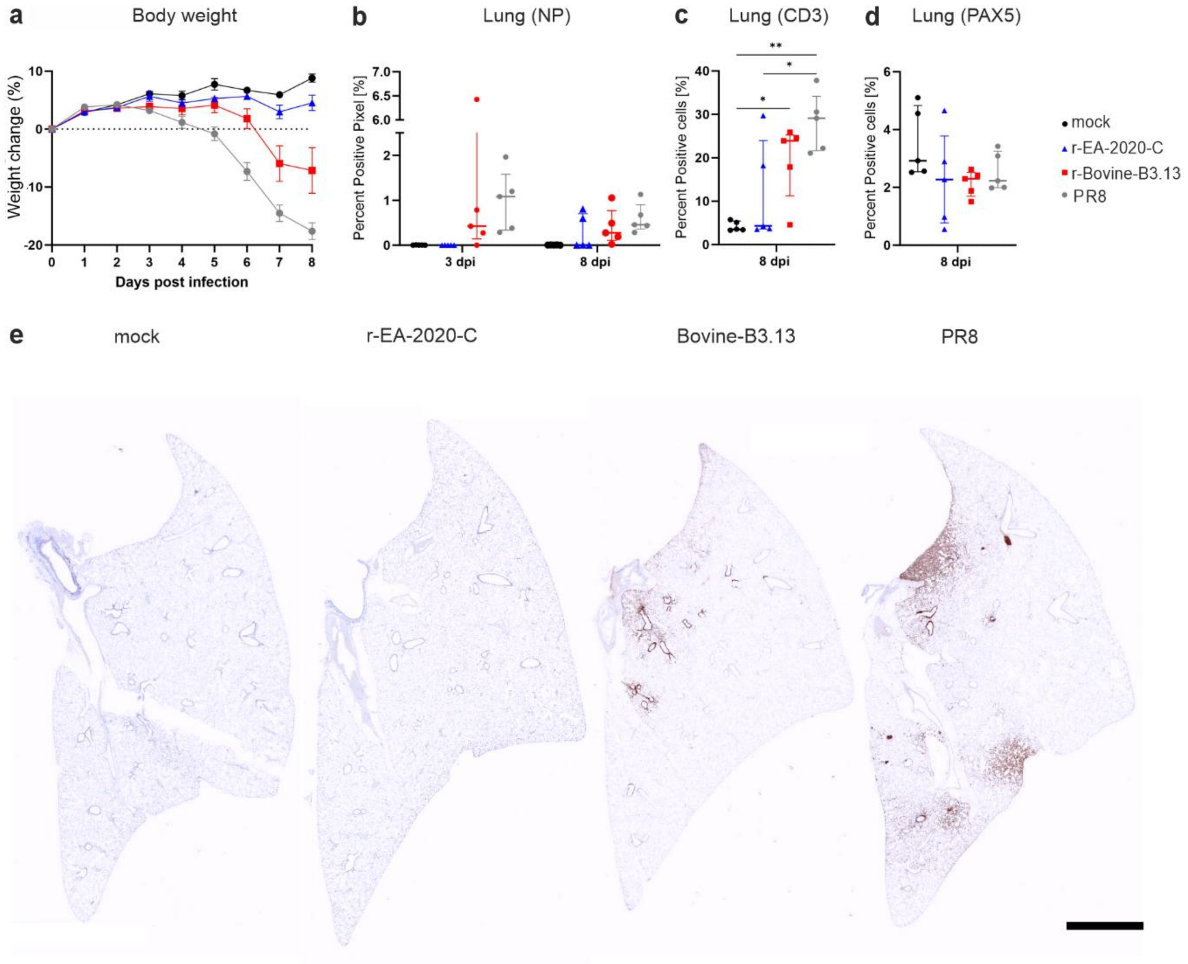


**Fig S4. Virulence of 2.3.4.4b reassortant viruses in an experimental mouse model.** Groups of 10 C57BL/6 mice were infected with 100 PFU of each recombinant virus, or mock-infected with PBS. Five mice were euthanized at 3 dpi and the remaining mice at 8 dpi. **a**, Weights were measured daily. **b-d**, Within the lung, the amount of NP (**b**), number of CD3 positive B cells (**c**) or Pax-5 positive T cells (**d**) was quantified in whole scanned slides of FFPE lung sections and calculated as positive pixel (NP, **b**) or positive cells (CD3, **c**; Pax-5, **d**) per lung area. **e**, Immunohistochemistry of lung sections collected from mock-infected and infected mice, r-EA-2020-C, r-Bovine-B3.13 and PR8, respectively, showing virus positive cells (NP) with brown signal in the bronchi and in the parenchyma (bar, 200 µm). Images shown are a representative example and sections from all 5 mice per cohort were imaged and analysed. In **b**) Statistical significance was determined using a repeated measures two-way ANOVA with Tukey's multiple comparison test (two-tailed; α= 0.05). Data in **c**) and **d**) were confirmed to be normally distributed using the Shapiro-Wilk Test and an ordinary one-way ANOVA with Tukey's multiple comparison test (two-tailed; α= 0.05) was used to compare groups.

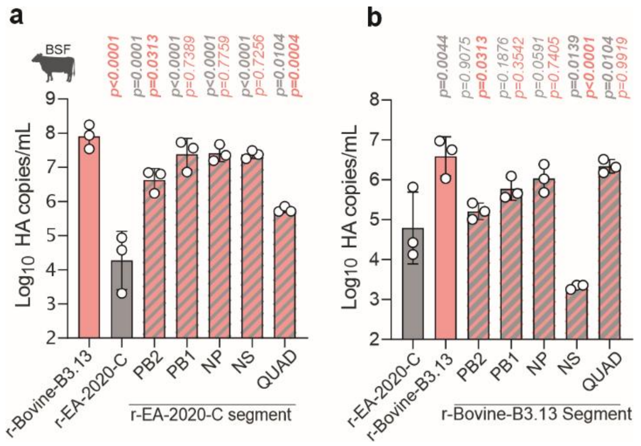


**Fig S5. Contribution of B3.13 internal genes to virus replication in bovine cells. a-b**, BSF cells were infected with 2:6 viruses bearing internal gene reassortant constellations between the ancestral r-EA-2020-C virus and r-Bovine-B3.13. The PB2, PB1, NP or NS segments, or all four ('QUAD'), were swapped reciprocally between the two. Cells were infected at an MOI of 0.0005 genome copies/cell and viral load in the supernatant at 24h post infection was determined by RT-qPCR as genome copies/mL. Data are mean +/- s.d. of three independent experiments. Each data point represents the mean of two technical duplicates. Data were log-transformed and confirmed to be normally distributed using the Shapiro-Wilk Test. Multiple comparisons between all viruses were performed using an ordi nary one-way ANOVA with Tukey's multiple comparison test (two-tailed; α = 0.05). Only com parisons relative to controls are shown. P-values in bold indicate statistical significance. (**a**) Created in BioRender. Bakshi, S. (2025) https://BioRender.com/xc6ow4u

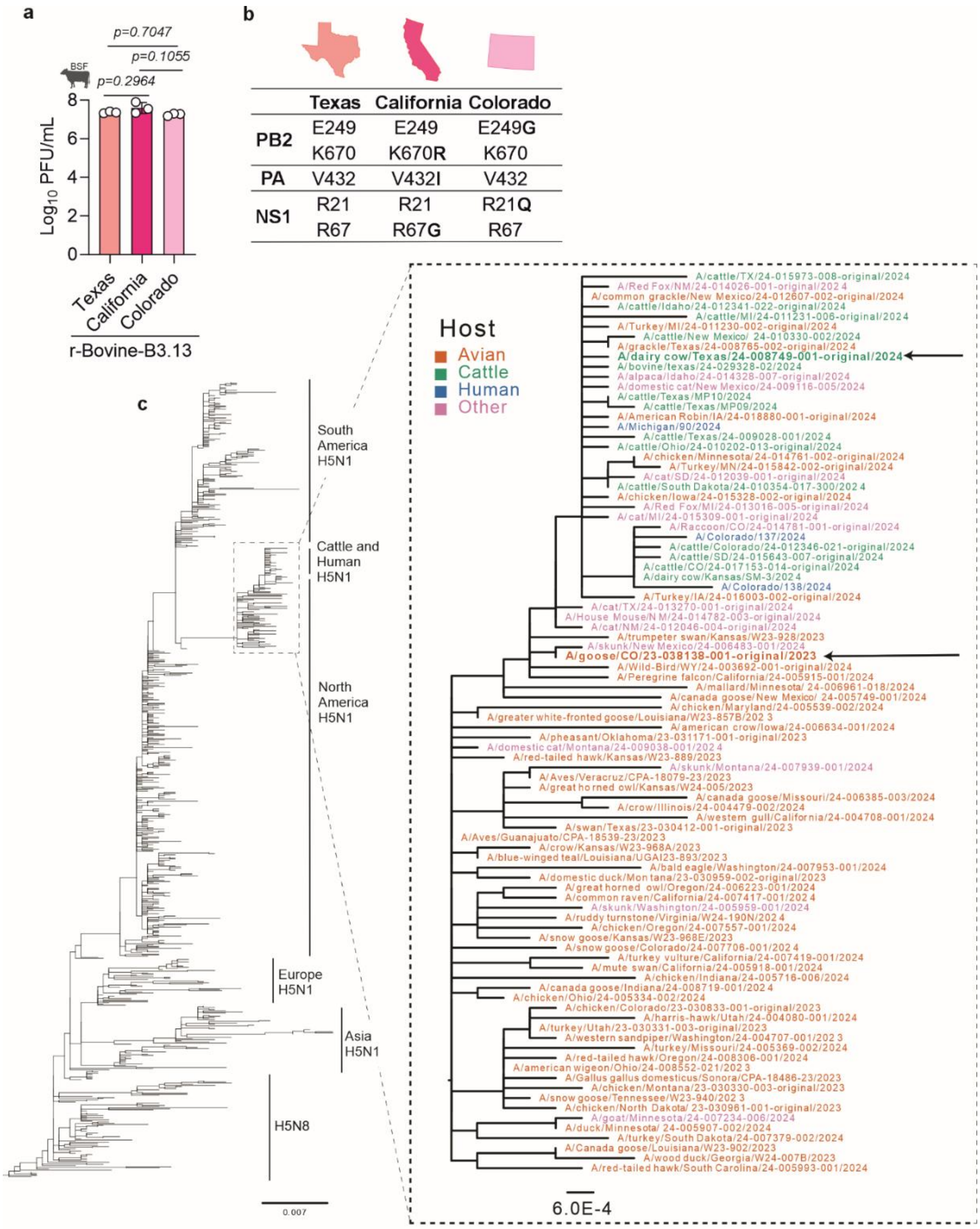


**Figure S6. Evolution and Replication of r-Bovine-B3.13 viruses that evolved during the outbreak. a**, Replication of r-Bovine-B3.13 Texas, Colorado and California virus strains in BSF cells at 24 hpi. Cells were infected with an MOI of 0.001 PFU/mL and virus titres in the supernatant were determined by plaque assay on MDCK cells. Data were log-transformed and assessed for normality using the Shapiro-Wilk Test. Statistical comparisons between all viruses were performed using an ordinary one-way ANOVA with Tukey's multiple comparison test (Two-tailed; α = 0.05). P values in bold indicate significance **b**, Table summarizing the amino acid substitutions acquired during the evolution of Bovine-B3.13 in dairy cattle throughout the outbreak. **c**, Maximum likelihood phylogeny of H5N1 HA de-duplicated amino acid sequences focusing on the evolution of the European and American 2.3.4.4b lineages. The part of the tree associated to the outbreak in dairy cattle is expanded and the tips are coloured according to the host. The tree was reconstructed usin g IQ-TREE2 with the best model selected by BIC (Bayesian Information Criterion). (**a-b**) Created in BioRender. Bakshi, S. (2025) https://BioRender.com/uagfptf

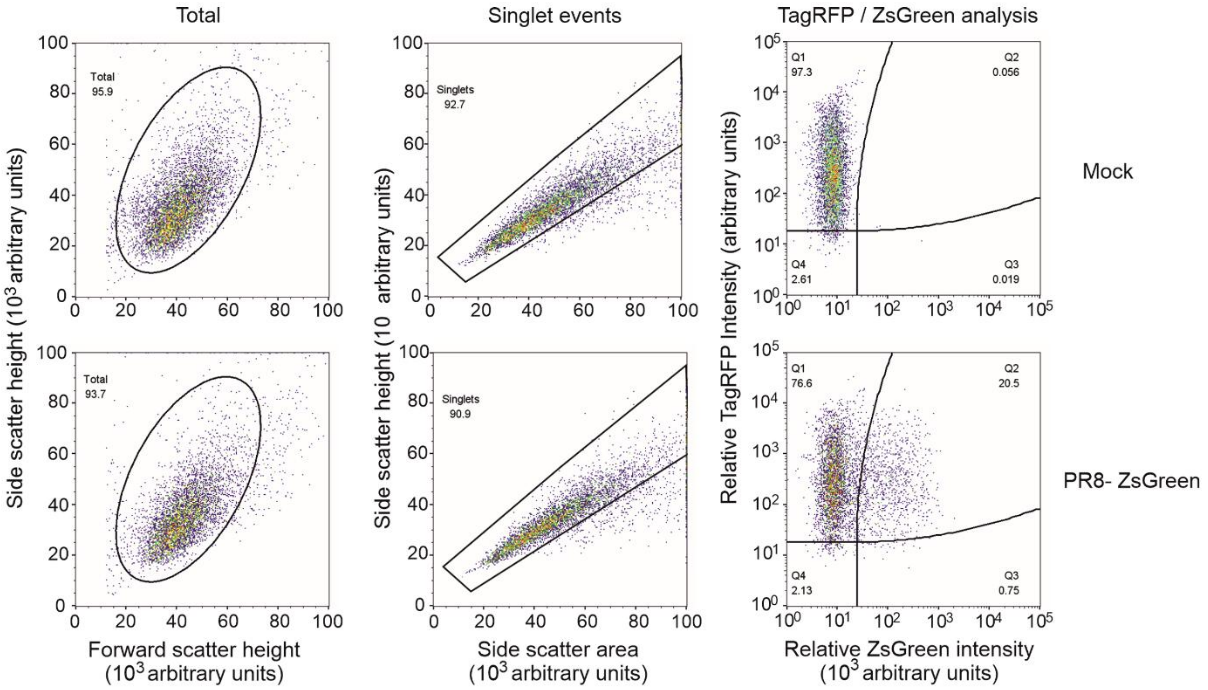


**Fig S7. Example flow cytometry gating of an IAV-ZsGreen titration on A549-SCRPSY-Mx1 cells in figure 6d.** Population gating was performed on FlowJo. Shown is an example of A549-Hs. MxA expressing cells either mock-infected (top row) or infected with PR8-ZsGreen (bottom row). A 'total' cell population (leftmost plots) was initially gated using forward and side scatter height intensity with a threshold applied to the forward scatter height to remove false events from debris or other particles. Single cells from this population (middle plots) were gated using side scatter height and area. Relative TagRFP (expressed by the SCRPSY-Mx1 lentiviral vector) and ZsGreen (expressed following IAV-ZsGreen virus infection) intensities were measured from this single cell population. A mock-infected sample was used to set the ZsGreen thresholding to gate for ZsGreen-positive events. The Q2 and Q3 plots were summed to calculate IAV infection percentage. The numerical values in each quadrant of each plot represents the percentage of cells in that population. This gating strategy was applied to flow cytometry data presented in Fig 6d.
