# Supplementary material for: The potential of H5N1 viruses to adapt to bovine cells varies throughout evolution
Source: Nat Commun. 2025 Dec 15;16:11042. doi: 10.1038/s41467-025-67234-1 (PMC12706089; doi:10.1038/s41467-025-67234-1)
Supplement: Supplementary file 2 — Description Of Additional Supplementary File [file 41467_2025_67234_MOESM2_ESM.pdf]

Description of Additional supplementary file

Supplementary data 1:

Sequences were prepared and analysed using DNA Dynamo and only amino acid differences relative to r-Bovine-B3.13 are shown. Residues are coloured according to charge. Positively charged (K/R/H) blue, negatively charged (D/E) orange, nonpolar residues (G/A/V/C/P/L/I/M/W/F) yellow, and polar residues (S/T/Y/N/Q) green.
