# Supplementary material for: The potential of H5N1 viruses to adapt to bovine cells varies throughout evolution
Source: Nat Commun. 2025 Dec 15;16:11042. doi: 10.1038/s41467-025-67234-1 (PMC12706089; doi:10.1038/s41467-025-67234-1)
Supplement: Supplementary file 4 — Reporting summary [file 41467_2025_67234_MOESM4_ESM.pdf]

nature portfolio

Corresponding author(s): Pablo Murcia & Massimo Palmarini

Last updated by author(s): Nov 19, 2025

Reporting Summary

Nature Portfolio wishes to improve the reproducibility of the work that we publish. This form provides structure for consistency and transparency in reporting. For further information on Nature Portfolio policies, see our Editorial Policies and the Editorial Policy Checklist.

Statistics

For all statistical analyses, confirm that the following items are present in the figure legend, table legend, main text, or Methods section.

n/a	Confirmed	
☐	☒	The exact sample size (*n*) for each experimental group/condition, given as a discrete number and unit of measurement
☐	☒	A statement on whether measurements were taken from distinct samples or whether the same sample was measured repeatedly
☐	☒	The statistical test(s) used AND whether they are one- or two-sided<br>*Only common tests should be described solely by name; describe more complex techniques in the Methods section.*
☒	☐	A description of all covariates tested
☐	☒	A description of any assumptions or corrections, such as tests of normality and adjustment for multiple comparisons
☐	☒	A full description of the statistical parameters including central tendency (e.g. means) or other basic estimates (e.g. regression coefficient) AND variation (e.g. standard deviation) or associated estimates of uncertainty (e.g. confidence intervals)
☐	☒	For null hypothesis testing, the test statistic (e.g. *F*, *t*, *r*) with confidence intervals, effect sizes, degrees of freedom and *P* value noted<br>*Give P values as exact values whenever suitable.*
☒	☐	For Bayesian analysis, information on the choice of priors and Markov chain Monte Carlo settings
☒	☐	For hierarchical and complex designs, identification of the appropriate level for tests and full reporting of outcomes
☒	☐	Estimates of effect sizes (e.g. Cohen's *d*, Pearson's *r*), indicating how they were calculated

Our web collection on statistics for biologists contains articles on many of the points above.

Software and code

Policy information about availability of computer code

Data collection	Western blotting data were collected on an Odyssey CLx infrared imaging system (LICOR CLX-2789)<br>qPCR assays were performed on an Applied Biosystems™ 7500 Fast Real-Time PCR machine<br>Guava InCyte 3.3 (https://www.merckmillipore.com/GB/en/20130828_204624?ReferrerURL=https%3A%2F%2Fwww.google.com%2F&bd=1) was used to acquire flow cytometry data.<br>Influenza A virus sequences were retrieved from the NCBI Entrez databases using the Influenza A virus taxonomic ID with an in-house Python tool to access the E-utilities API.<br>Tissue slides were digitised and scanned at 20x magnification using the Aperio Versa 8 Slidescanner and Aperio Versa 1.0.4.125 software (Leica Biosystems)<br>Confocal microscopy images were captured with a Zeiss LSM 710 Confocal Microscope (Zeiss, Wetzlar, Germany).<br>Luciferase activity was collected on a Promega GloMax Luminometer

Data analysis	GraphPad Prism 10 (https://www.graphpad.com/scientific-software/prism/) was used for plotting graphs and statistical analyses.<br>qPCR data was analysed on Applied Biosystems QuantStudio Design & Analysis Software 2.6.0.<br>FlowJo (https://www.flowjo.com) was used to analyze flow cytometry data using the most up-to-date software available.<br>Western blotting data were analyzed on Image Studio Lite https://www.licor.com/bio/image-studio-lite/)<br>To analyze Sanger sequencing data, DNA Dynamo software (https://www.bluetractorsoftware.com) was used.<br>The MMseqs2 software tool v15.6f452 was used to cluster sequences as described fully in Materials and Methods. MAFFT v7.453 was used to align sequences as described in Materials and Methods. Maximum likelihood trees were obtained with IQ-tree v2.1.2 under the best-fit model and plotted in RStudio using ggtree v3.12.0.<br>Images of digitised slides were captured using the Aperio ImageScope software v12.4.3.5008 (Leica Biosystems). Positive cell detection and quantification of CD3 and Pax-5, as well as positive pixel quantification of NP, were performed using QuPath software (version 0.4.3).

For manuscripts utilizing custom algorithms or software that are central to the research but not yet described in published literature, software must be made available to editors and reviewers. We strongly encourage code deposition in a community repository (e.g. GitHub). See the Nature Portfolio guidelines for submitting code & software for further information.

Data

Policy information about availability of data

All manuscripts must include a data availability statement. This statement should provide the following information, where applicable:

- Accession codes, unique identifiers, or web links for publicly available datasets
- A description of any restrictions on data availability
- For clinical datasets or third party data, please ensure that the statement adheres to our policy

The underlying data generated in this study have been deposited in the University of Glasgow Enlighten open access database under accession code 10.5525.gla.researchdata.2087 (doi: http://dx.doi.org/10.5525.gla.researchdata.2087)

Research involving human participants, their data, or biological material

Policy information about studies with human participants or human data. See also policy information about sex, gender (identity/presentation), and sexual orientation and race, ethnicity and racism.

Reporting on sex and gender	N/A
Reporting on race, ethnicity, or other socially relevant groupings	N/A
Population characteristics	N/A
Recruitment	N/A
Ethics oversight	N/A

Note that full information on the approval of the study protocol must also be provided in the manuscript.

Field-specific reporting

Please select the one below that is the best fit for your research. If you are not sure, read the appropriate sections before making your selection.

☒ Life sciences ☐ Behavioural & social sciences ☐ Ecological, evolutionary & environmental sciences

For a reference copy of the document with all sections, see nature.com/documents/nr-reporting-summary-flat.pdf

Life sciences study design

All studies must disclose on these points even when the disclosure is negative.

Sample size	Sample sizes were calculated assuming similar standard deviation as compared to historic controls to detect a difference of 5% in weight loss. This produced required sample sizes of 4 per group and so 5 mice were used per group to ensure sufficient power in the account of any unexpected losses.
Data exclusions	One recombinant virus generated in this study was determined to be the incorrect virus following analysis by sequencing and therefore all data associated with this was removed. Any ex vivo mammary tissue samples that had clear signs of bacterial contamination were removed.
Replication	All experimental data described in this manuscript were generated from at least three biological replicates which is standard in the field, except data associated with in vivo or ex vivo systems which are detailed in the respective figure legends and puromycin labelling experiments which were n =2.

Randomization	This study did not use subjects that required randomization
Blinding	Investigators were blinded to mouse group allocation during data collection. Investigators were unblinded during analysis as group identity was necessary to be known to calculate weight changes associated with each virus. Image histology scoring was conducted using software to ensure unbiased quantification. All other data collected in this study were objective, with no qualitative or subjective assessments, and therefore blinding was not applied.

Reporting for specific materials, systems and methods

We require information from authors about some types of materials, experimental systems and methods used in many studies. Here, indicate whether each material, system or method listed is relevant to your study. If you are not sure if a list item applies to your research, read the appropriate section before selecting a response.

Materials & experimental systems

n/a	Involved in the study
☐	☒ Antibodies
☐	☒ Eukaryotic cell lines
☒	☐ Palaeontology and archaeology
☐	☒ Animals and other organisms
☒	☐ Clinical data
☒	☐ Dual use research of concern
☒	☐ Plants

Methods

n/a	Involved in the study
☒	☐ ChIP-seq
☐	☒ Flow cytometry
☒	☐ MRI-based neuroimaging

Antibodies

Antibodies used	The primary antibodies used in this study are: PB2 - GeneTex (GTX125926), NP - MRC PPU Reagents and Services, Dundee (DA183, 5th Bleed), NS1 – (MRC PPU Reagents and Services, Dundee (DA182, 2nd Bleed), pSTAT1 (Tyr701) - Cell Signaling (9167S), IFIT1 - Origene (TA500948), RSAD2 - Proteintech (28089-1-AP), GAPDH - Cell Signaling (2118S), alpha-tubulin - Proteintech (66031-1-Ig), beta-actin - Proteintech (66009-1-Ig), Mx1 - Proteintech (13750-1-AP) and puromycin (Millipore; MABE343). A Mx1 antibody raised in mice was kindly provided by Georg Kochs (University Medical Centre, Freiburg, Germany). Secondary antibodies used: Anti-rabbit IgG (H+L) (DyLight 800 4X PEG Conjugate) Cell Signaling (5151S), Anti-mouse IgG (H+L) (DyLight 680 Conjugate) Cell Signaling (5470S) and Anti-sheep IgG (H+L) (Alexa FlourTM 680) Thermo Fisher Scientific (A21102).<br><br>All primary antibodies were used at 1:1000 except anti-GAPDH (1:10 000). All secondary antibodies were used at 1:10 000 except anti-mouse and anti-sheep (1:20 000)
Validation	GTX125926 - Validation shown by manufacturer for WB, ICC/IF and IP comparing mock infected and H1N1 infected cells. Cited 81 times.<br>DA183 - Validated for WB, IF and ELISA at the CVR by comparing mock infected and PR8 H1N1 infected MDCKs.<br>DA182 - Validated for WB, IF and ELISA at the CVR by comparing mock infected and PR8 H1N1 infected MDCKs.<br>9167S - Validation shown by manufacturer for several techniques (WB, IP, IHC, IF, ChIP). Validated by comparison of mock-treated cells compared to cells treated with IFN-alpha or IFN-gamma. Migrates at the expected molecular weight. Cited 1152 times.<br>TA500948 - Validated by manufacturer for FC, WB, IF and IHC. Cited 8 times.<br>28089-1-AP - Validated by the manufacturer using WB and IHC. Migrates at the expected molecular weight. Cited 18 times which included a KD validation.<br>2118S - Validated by the manufacturer for several techniques (WB, IHC, IF). Migrates at the expected molecular weight. Cited 8867 times.<br>66031-1-Ig - Validated by the manufacturer for several techniques (WB, IHC, IF/ICC, FC, IP, ELISA). Migrates at the expected molecular weight. Cited 1466 times.<br>66009-1-Ig - Validated by the manufacturer for several techniques (WB, IHC, IF/ICC, FC, IP, ChIP, ELISA). Migrates at the expected molecular weight. Cited 8241 times.<br>13750 -1-AP - Validated by the manufacturer for several techniques (WB, IP, IHC, IF/ICC, FC). Mx1 is observed only upon IFN treatment compared to untreated cells. Migrates at the expected molecular weight.<br>MABE343 - Validated by the manufacturer for FACS, ICC, IF, IHC and IP. Cited 657 times.<br>Mx1 antibody - Validated by WB. Mx1 is observed only upon IFN treatment compared to untreated cells. Migrates at the expected molecular weight. (Referece - Flohr, F., Schneider-Schaulies, S., Haller, O., Kochs, G., 1999. The central interactive region of human MxA GTPase is involved in GTPase activation and interaction with viral target structures. FEBS Lett. 463, 24–28. https://doi.org/10.1016/s0014-5793(99)01598-7)

Eukaryotic cell lines

Policy information about [cell lines and Sex and Gender in Research]

Cell line source(s)	MDCK, A549, and HEK-293T were from ATCC.<br>Bovine skin fibroblasts were isolated from primary tissue and immortalised with hTERT as described in doi: 10.1128/mbio.00101-23<br>Bovine nasal and udder fibroblasts were isolated from tissue from the abattoir using standard protocols.

Authentication	HEK-293T and A549 cells were authenticated using a commercial cell line authentication service (Eurofins)
Mycoplasma contamination	All cell lines used in this study were tested for mycoplasma contamination upon introduction into the laboratory and were confirmed to be negative. Routine testing was not performed.
Commonly misidentified lines (See ICLAC register)	HEK-293T (authenticated)

Animals and other research organisms

Policy information about studies involving animals; ARRIVE guidelines recommended for reporting animal research, and Sex and Gender in Research

Laboratory animals	Mice (male, 8 week old C57BL/6) were obtained from Charles River Laboratories (UK)
Wild animals	N/A
Reporting on sex	Male mice were used as they show greater clinical signs and weight loss during PR8 infection making differential comparisons between strains more sensitive (doi: 10.3389/fimmu.2018.01747)
Field-collected samples	*For laboratory work with field-collected samples, describe all relevant parameters such as housing, maintenance, temperature, photoperiod and end-of-experiment protocol OR state that the study did not involve samples collected from the field.*
Ethics oversight	All animal work was in accordance with the animal ethics and welfare committee at the University of Glasgow and the United Kingdom Home Office regulations (ASPA, 1986, PPL PP4085778).

Note that full information on the approval of the study protocol must also be provided in the manuscript.

Plants

Seed stocks	*Report on the source of all seed stocks or other plant material used. If applicable, state the seed stock centre and catalogue number. If plant specimens were collected from the field, describe the collection location, date and sampling procedures.*
Novel plant genotypes	*Describe the methods by which all novel plant genotypes were produced. This includes those generated by transgenic approaches, gene editing, chemical/radiation-based mutagenesis and hybridization. For transgenic lines, describe the transformation method, the number of independent lines analyzed and the generation upon which experiments were performed. For gene-edited lines, describe the editor used, the endogenous sequence targeted for editing, the targeting guide RNA sequence (if applicable) and how the editor was applied.*
Authentication	*Describe any authentication procedures for each seed stock used or novel genotype generated. Describe any experiments used to assess the effect of a mutation and, where applicable, how potential secondary effects (e.g. second site T-DNA insertions, mosiacism, off-target gene editing) were examined.*

Flow Cytometry

Plots

Confirm that:

☒ The axis labels state the marker and fluorochrome used (e.g. CD4-FITC).

☒ The axis scales are clearly visible. Include numbers along axes only for bottom left plot of group (a 'group' is an analysis of identical markers).

☒ All plots are contour plots with outliers or pseudocolor plots.

☒ A numerical value for number of cells or percentage (with statistics) is provided.

Methodology

Sample preparation	A549 cells (CLA authenticated by Eurofins Genomics Ltd) in 96-well plates were infected with a serial dilution of IAV-ZsGreen, or mock infected, for 7h. Cells were washed with PBS, trypsinised to dissociate from the plasticware, resuspended in growth medium, and then mixed 1:1 with 4% formaldehyde (to achieve final concentration of 2% formaldehyde).
Instrument	In this study, two biological repeats were run on a Luminex Guava easyCyte and one biological repeat was run on a Millipore guava easyCyte HT using Guava InCyte software.
Software	Collection of flow cytometry data used GUAVA InCyte software. Analysis was performed on FlowJo version 10.
Cell population abundance	The mean event count of post-sort samples from an example 96-well plate used in this study was 4474 events (with SD 1117) and median event count of 4553
Gating strategy	Population gating was performed on FlowJo version 10. A 'total' cell population was initially gated using forward and side

Gating strategy

scatter height intensity with a threshold applied to the forward scatter height to remove false events from debris or other particles. Single cells from this population were gated using side scatter height and area. Relative TagRFP (expressed by the SCRPSY-Mx1 lentiviral vector) and ZsGreen (expressed following IAV-ZsGreen virus infection) intensities were measured from this single cell population. A mock-infected sample was used to set the thresholding of ZsGreen-positive events.

☒ Tick this box to confirm that a figure exemplifying the gating strategy is provided in the Supplementary Information.
